# Seismic history of western Anatolia during the last 16 kyr determined by cosmogenic ^36^Cl dating

**DOI:** 10.1186/s00015-022-00408-x

**Published:** 2022-02-17

**Authors:** Nasim Mozafari, Çağlar Özkaymak, Ökmen Sümer, Dmitry Tikhomirov, Bora Uzel, Serdar Yeşilyurt, Susan Ivy-Ochs, Christof Vockenhuber, Hasan Sözbilir, Naki Akçar

**Affiliations:** 1 Institute of Geological Sciences, University of Bern, Baltzerstrasse 1+3, 3012 Bern, Switzerland; 2 Department of Geological Engineering, Afyon Kocatepe University, Ahmet Necdet Sezer Kampusü Gazligöl Yolu, 03200 Afyonkarahisar, Turkey; 3 Earthquake Research and Application Center of Afyon Kocatepe University, Ahmet Necdet Sezer Kampusü Gazligöl Yolu, 03200 Afyonkarahisar, Turkey; 4 Geological Engineering Department, Dokuz Eylül University, 35160 İzmir, Turkey; 5 Department of Geography, University of Zurich, Winterthurerstrasse 190, 8057 Zurich, Switzerland; 6 Department of Geography, Ankara University, 06230 Ankara, Turkey; 7 Institute for Particle Physics, ETH Hönggerberg, Schafmattstrasse 20, 8093 Zürich, Switzerland; 8 Earthquake Research and Implementation Center, Dokuz Eylul University, 35160 İzmir, Turkey

**Keywords:** Fault scarp dating, Eastern Mediterranean, Recurrence interval, ^36^Cl exposure dating, Earthquake

## Abstract

Western Anatolia is one of the most seismically active regions worldwide. To date, the paleoseismic history of many major faults, in terms of recurrence intervals of destructive earthquakes, their magnitude, displacement, and slip rates is poorly understood. Regional crustal extension has produced major horst-graben systems bounded by kilometer-scale normal faults locally in carbonates, along which vertical crustal displacements occurred. In this study, we explore the seismic history of western Anatolia using ^36^Cl exposure dating through study of well-preserved carbonate normal fault scarps. To accomplish this, ^36^Cl concentrations in 214 samples from fault plane transects on the Rahmiye and Ören fault scarps were measured and compared with existing ^36^Cl measurements of 370 samples on five fault scraps in western Anatolia. At least 20 seismic events have been reconstructed over the past 16 kyr. The age correlation of the seismic events implies four phases of high seismic activity in western Anatolia, at around 2, 4, 6, and 8 ka. Slips are modeled ranging between 0.6 to 4.2 m per seismic event, but are probably the result of clustered earthquakes of maximum magnitude 6.5 to 7.1. While the average slip rates have values of 0.3 to 1.9 mm/yr, incremental slip rates of the faults range greater than 0.1 to 2.2 mm/yr, showing more activity mostly through late Holocene. Our finding reveals high capability of cosmogenic ^36^Cl dating to explore seismic behavior of active faults beyond the existing earthquake records.

**Supplementary Information:**

The online version contains supplementary material available at 10.1186/s00015-022-00408-x.

## Introduction

Long-term seismic modeling is a fundamental source for seismic hazard assessment, which is principally based on the estimation of crucial parameters namely earthquake recurrence intervals, magnitudes, and slip rates (e.g., Cluff & Cluff, 1984; Weldon et al., 2004; McCalpin, 2009; Wang, 2009; Salditch et al., 2020). These parameters are approximated using historical and instrumental earthquake data in combination with geological field observations. The earthquake recurrence interval is particularly an efficient aspect in seismic hazard assessment, specifically in tectonic settings where sporadic seismic activity is separated by dormant phases and the recurrence interval is constant through time, such as along continental intraplate faults (Camelbeeck et al., 2007; Crone et al., 2003; Leonard et al., 2007). Generally, the recurrence interval can be estimated based on the time period between major earthquakes or earthquake clusters either on a single fault or on several faults of the same tectonic setting (e.g., Sieh, 1981; Wallace, 1981); however, some paleoseismology studies show variable recurrence intervals along individual faults of a certain fault network and even on the same fault (Dawson et al., 2003; Grant & Sieh, 1994; Marco et al., 1996; Mouslopoulou et al., 2009; Nicol et al., 2009, 2016; Palumbo et al., 2004; Sieh et al., 1989; Weldon et al., 2004). In addition to the abovementioned essential factors, knowledge about the nature of the occurrence of seismic events, as an individual event or clustering earthquakes—a sequence of earthquakes of rather similar size occurring in a given area over a relatively short period of time, excluding aftershocks—carries a high value of importance in terms of vulnerability management for both seismic hazard and risk assessment (Salditch et al., 2020; Stein et al., 2017). Understanding of seismic behavior of an area by analyzing the pattern of clustered earthquakes provide more valuable insights into the earthquake hazard assessment than those depending on probability density function of recurrence intervals (Salditch et al., 2020).

Western Anatolia is such a vulnerable region, where earthquakes have frequently caused significant damage in urban areas since the antiquity (e.g., Ambraseys, 2009; Shebalin et al., 1974; Soysal et al., 1981). For example, 119 people were killed and 1053 injured, and many buildings were collapsed during the Samos–İzmir earthquake (Mw = 7.0) that occurred on October 30th, 2020 (Kandilli Observatory and Earthquake Research Institute, Boğaziçi University). In this vulnerable region, high seismic activity is the result of ruptures generated by normal faulting, which basically occur in E-W-trending horst-graben systems that formed in response to the approximately N-S extensional tectonic regime since the Early Miocene, including the Gediz-Alaşehir, Küçük Menderes, Büyük Menderes and Gökova graben (e.g., Çiftçi & Bozkurt, 2009; Emre & Sözbilir, 2007; Özkaymak et al., 2013; Sözbilir et al., 2011; Sümer, 2015; Sümer et al., 2013). In addition to historical and instrumental records, the occurrence of past ruptures is evidenced by heavy damage to several archaeological sites (e.g., Altunel, 1998; Yönlü et al., 2010). Moreover, several trench-based studies revealed past earthquakes, their timing, and the amounts of associated slip (e.g., Altunel et al., 2009; Özkaymak et al., 2011). Yet the seismic behavior of the western Anatolia major faults, in terms of recurrence intervals of destructive earthquakes, their magnitude, and displacement amount, is poorly constrained. The earliest recorded earthquake in the eastern Mediterranean and Middle East dates back to 2100 BC (Ambraseys, 2009; Shebalin et al., 1974; Soysal et al., 1981), whereas instrumental earthquakes have only been recorded since 1900. Furthermore, archaeoseismic studies, which focus on the effects of earthquakes on historical architecture, only record past earthquakes within the last 5–6 kyr (Caputo & Helly, 2008). However, for long-term seismic modeling, both a better temporal resolution of earthquakes and longer temporal record are required.

Besides trenching and archaeoseismic studies, the dating of carbonate fault scarps using cosmogenic ^36^Cl offers a direct and precise method to reconstruct past earthquakes by analyzing phases of activity and inactivity along a carbonate fault scarp height (e.g., Schlagenhauf et al., 2010). The presence of well-preserved normal fault scarps (i.e., with a minimum degree of weathering and erosion) in western Anatolia, which locally occur in calcareous rocks, makes this region well-suited for the use of cosmogenic ^36^Cl exposure dating in exploring the paleoseismicity. In this method, a normal fault surface is utilized as a geological marker to determine the earthquake horizons following temporal accumulation of cosmogenic ^36^Cl. Episodic seismic activity on the fault causes a discontinuous profile in the cosmogenic ^36^Cl concentrations, which allows the timing of past ruptures and their vertical slip magnitude to be defined. During the quiescence, in the part of a fault scarp, which is covered by colluvium, the accumulated cosmogenic ^36^Cl in the outermost few centimetres of the fault surface, form an exponential profile (Additional file 1: Fig. S1). Once an earthquake exposes the buried part of the fault surface, accumulation of cosmogenic ^36^Cl on the unshielded fault surface occurs at a higher rate than the time when this part of the fault surface was still buried. Consequently, following the occurrence of repeated earthquakes along a vertical profile on a fault scarp, ^36^Cl concentrations would theoretically display several cusps that indicate periods of high seismic activity and intervening approximate-convex curves, which reveal the phases of inactivity (e.g., Benedetti et al., 2002, 2003; Mozafari, et al., 2019; Tikhomirov et al., 2019) (Additional file 1: Fig. S1). Though, the modeling of the cosmogenic ^36^Cl accumulation along the fault scarp based on the measured nuclide concentrations reveals the earthquakes timing and corresponding dip-slip values. Vertical distance between two succeeding discontinuities shows the slip magnitude related to the seismic event that caused the episodic exposure of the fault surface (Additional file 1: Fig. S1).

**Fig. 1:**
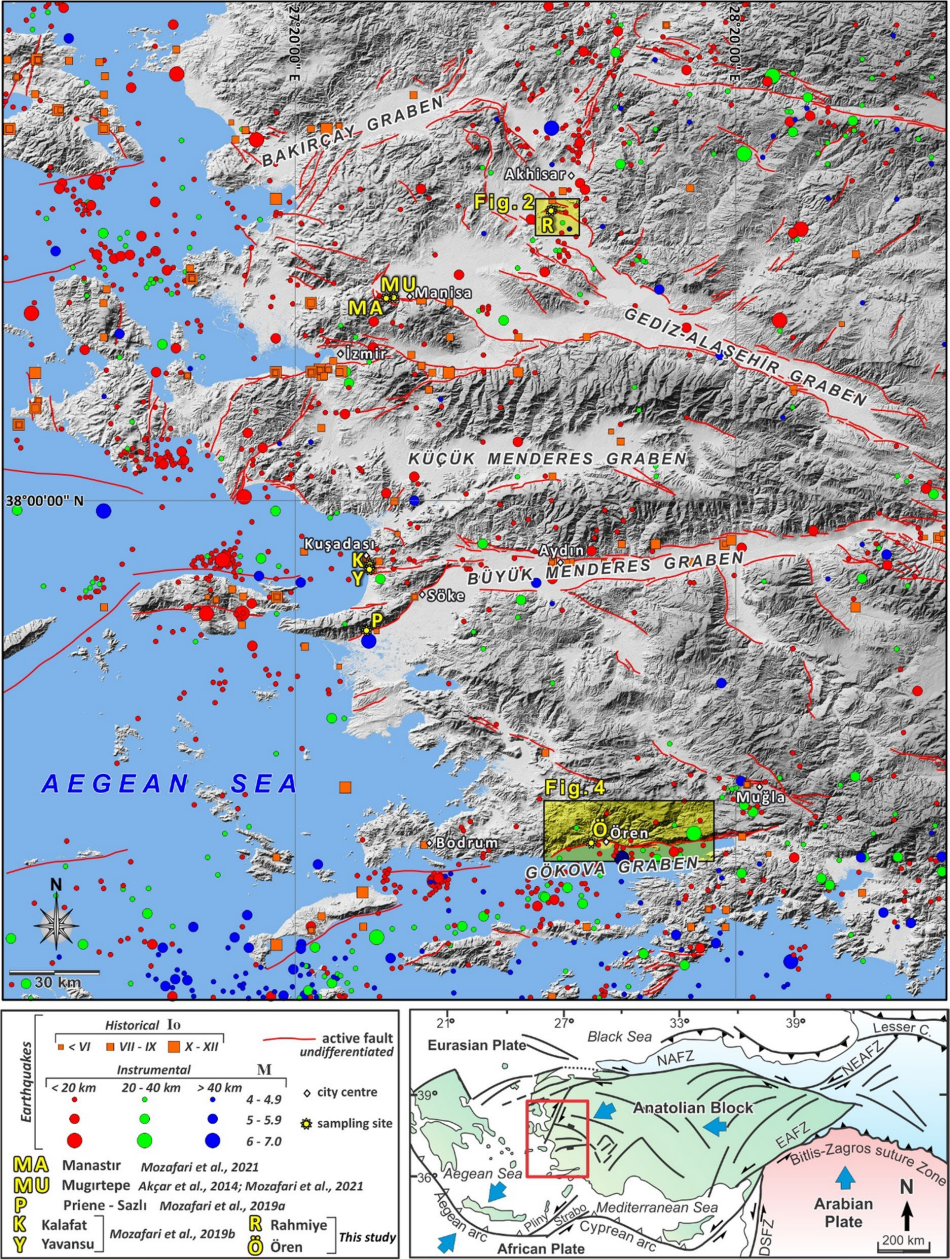
Seismotectonic map of Western Anatolia (active faults are compiled from Emre et al. (2018) and The Gre.Da.S.S. Working Group, Pavlides et al. (2014); the historical and instrumental earthquakes (1900–2016) are taken from Duman et al. (2016) and reference therein. Instrumental earthquakes (2016–2021) are obtained from Kandilli Observatory and Earthquake Research Institute. Locations of fault scarp studies are marked in yellow abbreviations

**Fig. 2:**
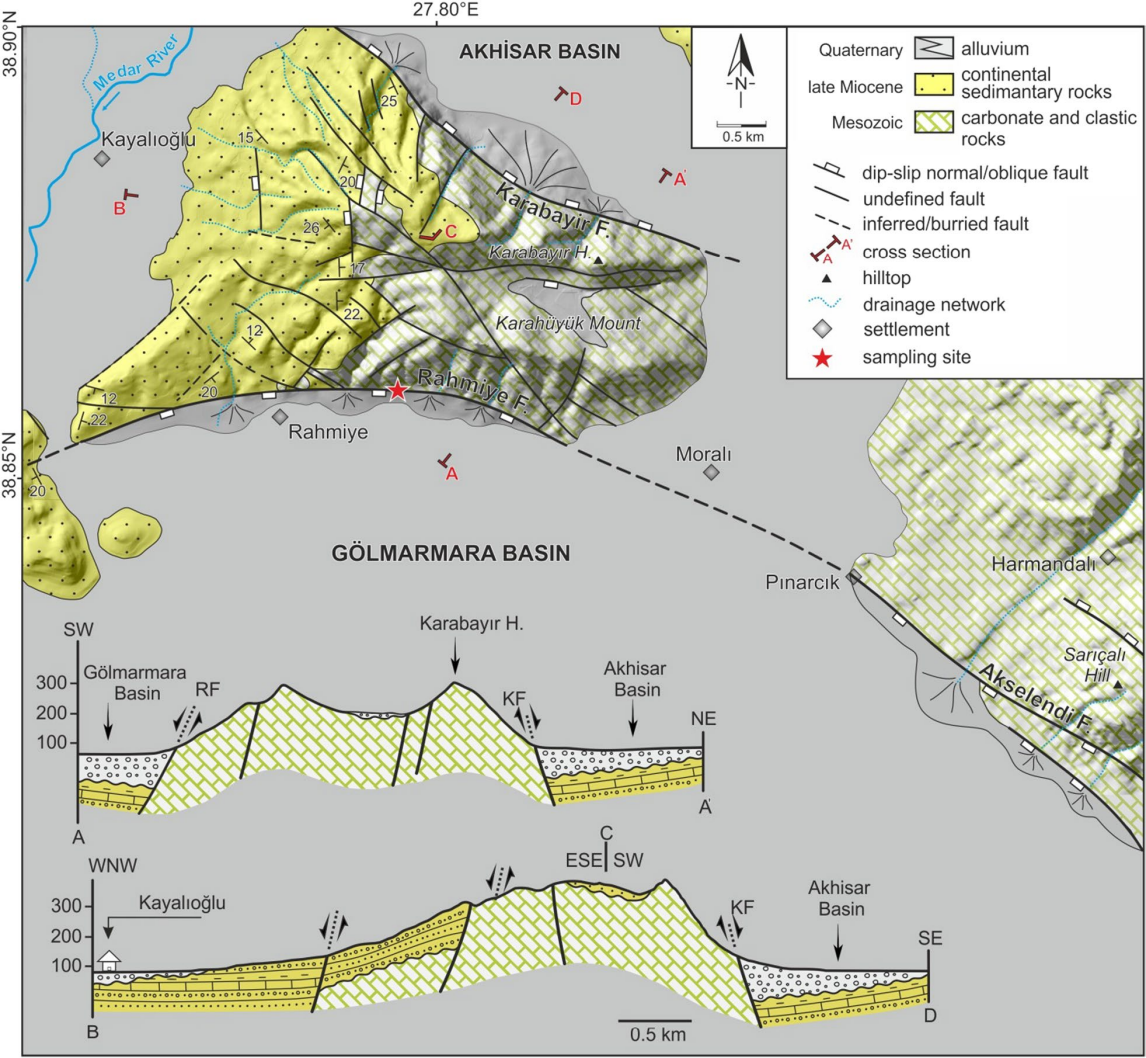
Geological map and cross sections of the northern border of the Gölmarmara Basin controlled by the Rahmiye fault, western end of the Gediz Graben. The star marks the sampling site. RF: Rahmiye fault, KF: Karabayır fault

**Fig. 3:**
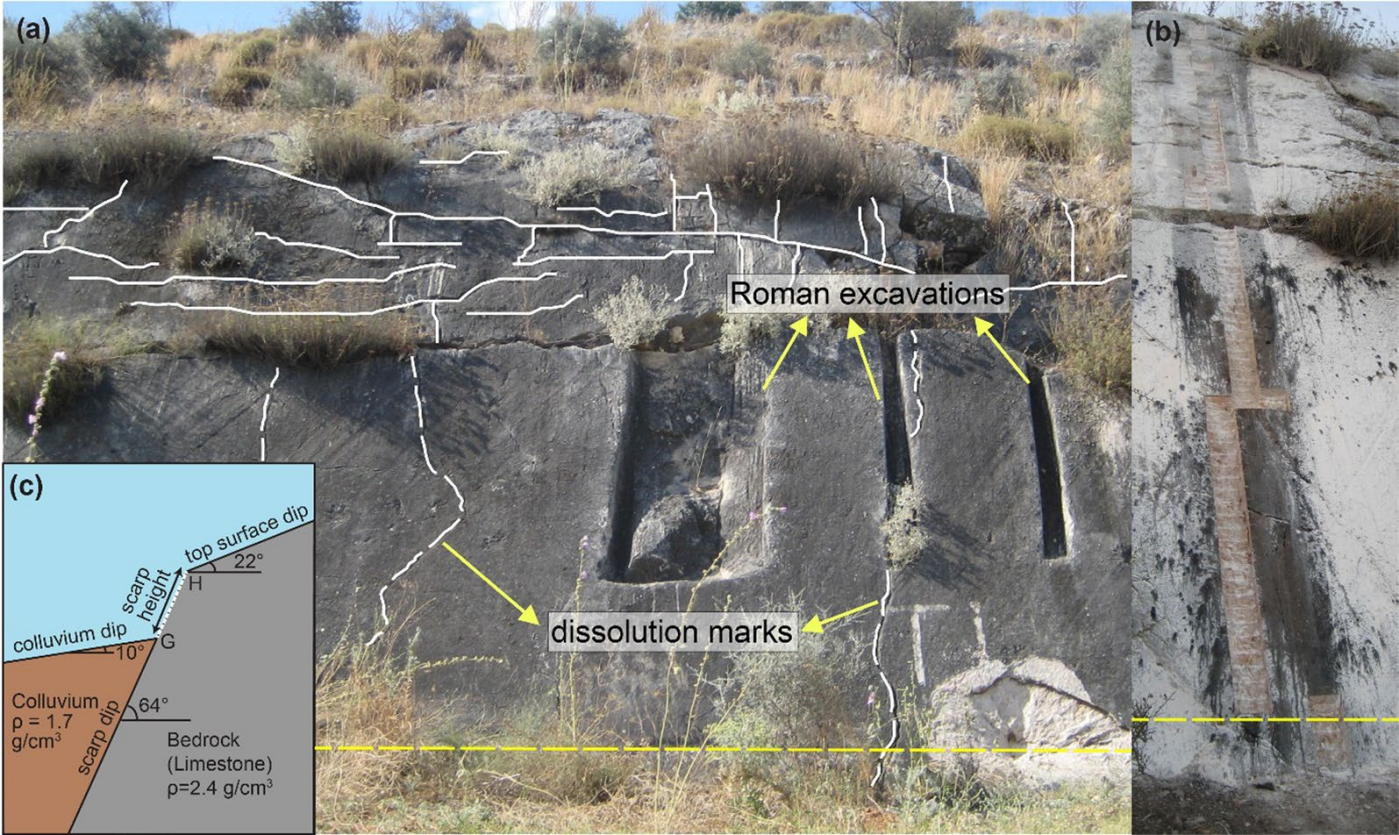
**a** Rahmiye fault scarp surface showing two patterns of weathering; solid lines in the upper section show horizontal and vertical fractures and dissolutions, whereas dashed lines represent vertical dissolution traces in the lower part of the fault surface; **b** the sampled profile of the Rahmiye fault scarp; and **c** Cartoon of Rahmiye fault showing important parameters of the fault scarp for modeling, including scarp height, scarp dip, colluvium dip, top surface dip and density of the bedrock and colluvium. White dashed line shows the sampled surface

**Fig. 4:**
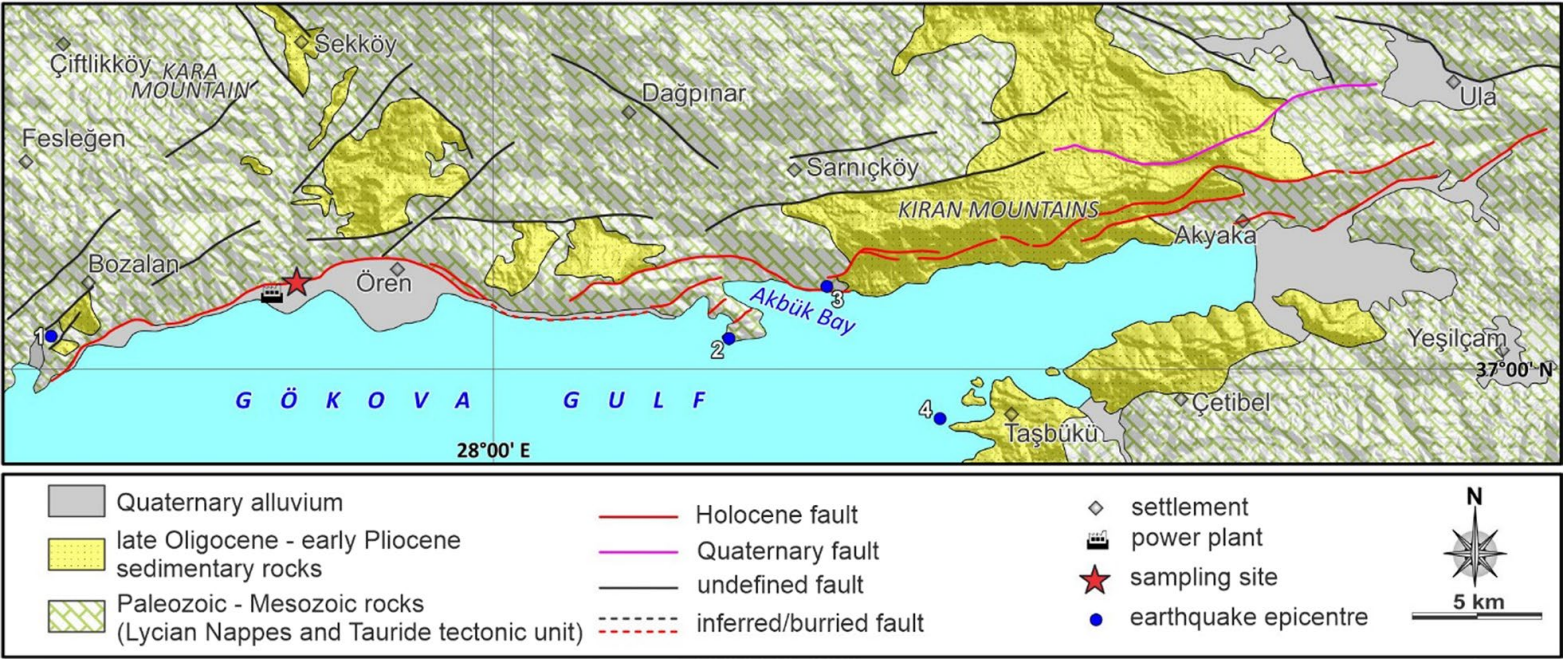
Simplified geological map of the Ören fault around the sampling site (modified after Duman et al., 2011; Gürer & Yılmaz, 2002; Gürer et al., 2013). Earthquake epicenters: (1) January 10, 2005 (Mw: 5.5) (United States Geological Survey National Earthquake Information Center; earthquake.usgs.gov/contactus/golden/neic.php); (2) April 25, 1959 (Mw: 5.9); (3) December 13, 1941 (Mw: 6.3); (4) May 23, 1941 (Mw: 5.4); (Tiryakioğlu et al., 2018 and references therein). The epicenters of the earthquakes from August 04, 2004 (Mw: 5.3) and December 20, 2004 (Mw: 5.3) are outside area of this map

**Fig. 5:**
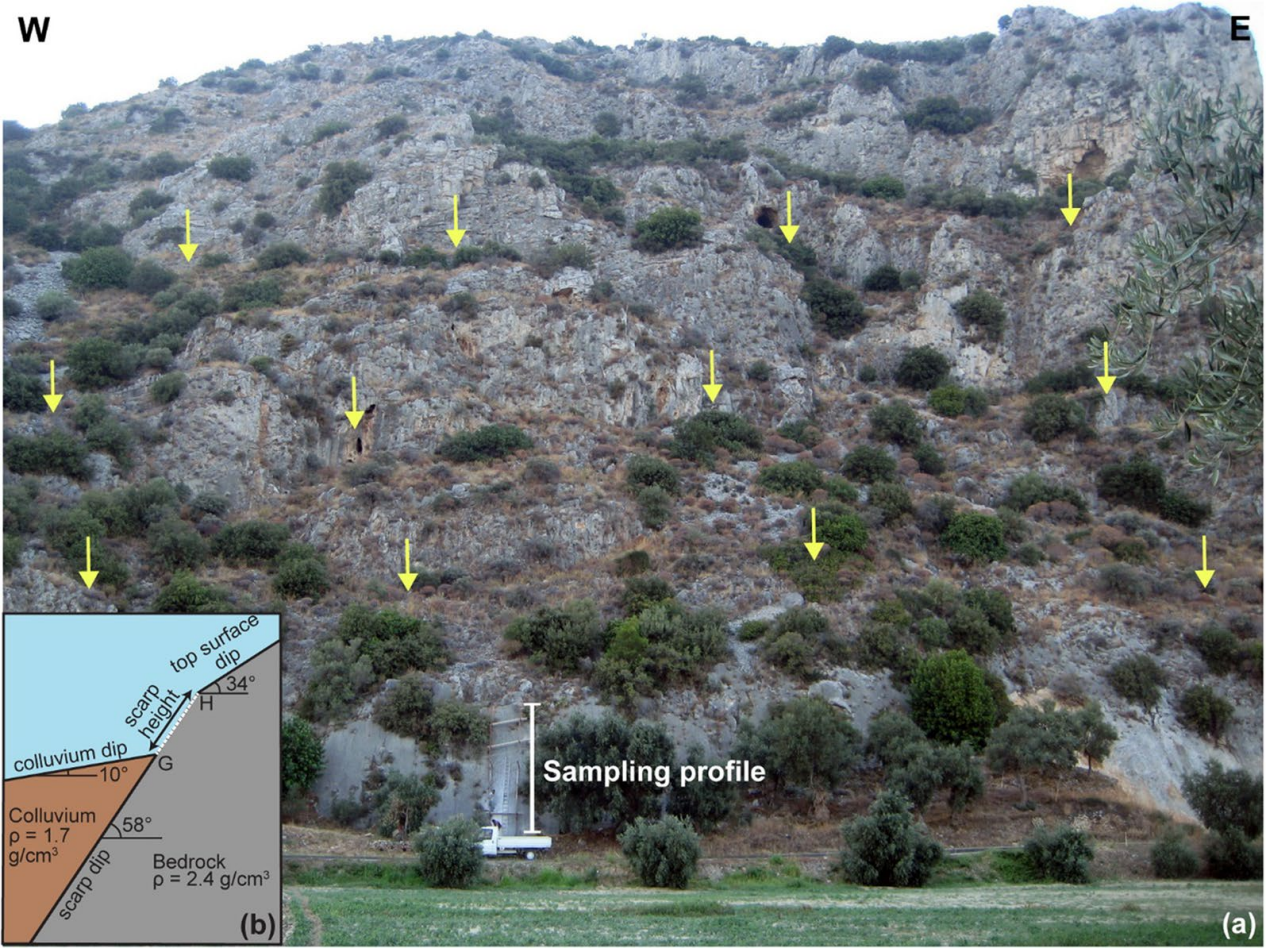
**a** Field view of the Ören fault scarp with the sampling strip on the lowermost of a series of scarps indicated by arrows; and **b** Cartoon of Ören fault showing important parameters of the fault scarp for modeling, including scarp height, scarp dip, colluvium dip, top surface dip and density of the bedrock and colluvium. White dashed line shows the sampled surface

In this study, we aim to provide data for seismic hazard mitigation in western Turkey by: (1) reconstructing past seismic events and determining the recurrence intervals of earthquakes by applying fault scarp dating using cosmogenic ^36^Cl to define the main phases of activity on the regional scale; (2) quantifying the slip values and assessing the plausibility of the modeled scenarios; and (3) estimating the incremental and average slip rates. To achieve these aims, we improved the seismic datasets of western Anatolia based on cosmogenic ^36^Cl by studying two new major faults in detail: the Rahmiye fault in the Gediz-Alaşehir Graben and the Ören fault along the Gökova Graben (Figs. 1, 2, 3, 4 and 5). These faults are important in terms of being considered as two key faults on the southern and northern boundaries of the western Anatolian extensional regime, which enable us to enlarge the seismic database yielded using fault scarp dating through the entire western Anatolia within the time and financial constraints (Akçar et al., 2012; Mozafari, et al., 2019; Mozafari, et al., 2019, 2021). We reconstructed a chronological framework for these well-preserved normal fault scarps by measuring cosmogenic ^36^Cl concentrations in 214 samples (Fig. 1). From the measured ^36^Cl concentrations, we modeled several significant seismic events using a Matlab® code based on the Monte Carlo method (Tikhomirov, et al., 2019). By means of cosmogenic ^36^Cl chronology, we computed the amount of dip-slip component of displacement on the fault surface by considering the most plausible events and estimated the incremental and average slip rates. In addition, we reevaluated recently published data of 370 cosmogenic ^36^Cl concentrations from the Manastır and Mugırtepe faults in the Gediz-Alaşehir Graben (Akçar et al., 2012; Mozafari et al., 2021), and the Kalafat, Yavansu, and Priene-Sazlı faults in the Büyük Menderes Graben (Mozafari et al., 2019, 2019) using the same approach. Slip rate values of the last three faults above are recalculated with regards to modeled throw/maximum vertical displacement instead of considering dip-slip values on the fault surface. Moreover, the elapsed times since the most recent earthquakes to the current time are taken into account. We finally established a comprehensive regional seismic history.

## Existing cosmogenic ^36^Cl chronology of seismic activity and the faults recorded seismic background in western anatolia

For the past decade, we focused on the reconstruction of destructive seismic events beyond the temporal range of existing archaeoseismic, historical and instrumental data of normal fault scarps in the major graben systems in western Anatolia (Akçar et al., 2012; Tikhomirov, 2014; Tikhomirov et al., 2019; Mozafari, et al., 2019; Mozafari, et al., 2019, 2021). The outcome of our studies is briefly outlined below.

### Gediz-Alaşehir Graben

The WNW-ESE-trending Gediz-Alaşehir Graben is approximately 150 km long and is the northern most structural element in the western Anatolian extensional province (Fig. 1). The Menderes Massif constitutes the bedrock of the Gediz-Alaşehir Graben with its horst blocks having a maximum elevation of 2000 m (Bozkurt and Sözbilir, 2006; Çiftçi & Bozkurt, 2009). The floor of the graben is composed of Miocene to Quaternary sediments (Çiftçi & Bozkurt, 2009). Well preserved scarps of the Manastır and Mugırtepe faults in the west part of the graben system and westernmost part of the approximately 35 km long active Manisa Fault Zone (Emre et al., 2018; Özkaymak et al., 2011, 2013) have been previously dated using cosmogenic ^36^Cl (Akçar et al., 2012; Mozafari et al., 2021) to investigate the seismic history of Manisa Fault Zone (Fig. 1). Several major historical earthquakes were recorded in this area such as the earthquake of 17 AD that caused severe damage to many ancient cities, including cities within the Manisa Basin, with evidence of surface rupture (Ambraseys, 1988; Guidoboni et al., 1994). An additional earthquake with an intensity of VIII struck the ancient cities of Magnesia and Ephesus in 44 AD (Ergin et al., 1967; Soysal et al., 1981). It should be noted that none of the historical earthquakes in this area have been directly attributed to the Manisa fault. The largest instrumental earthquake recorded close to Manisa occurred on January 28th, 1994, with Mw 5.2 or 5.4 (Tan et al., 2008; United States Geological Survey National Earthquake Information Center) (Fig. 2).

The NW–SE trending Manastır fault escarpment constitutes the southwestern splay of the Manisa Fault Zone and is approximately 4.5 km long and up to 140 m high. The footwall of the fault is composed of Mesozoic limestone bedrock, whereas the hangingwall is mantled by Holocene colluvium. Manastır is considered as a master fault with basinward migration growth of several secondary parallel faults, namely Mugırtepe. To model the paleoseismicity, a total of 87 fault scarp samples were collected for ^36^Cl analyses from multiple sampling strips covering 7 m of the scarp along a ca. 12 m long section of the Manastır fault (Tikhomirov, 2014). Due to intense weathering, the upper section was not sampled. Two seismic events were reconstructed at ca. 3.5 and 2 ka, with ca. 3.3 and 3.6 m of slip, and 2.2 and 1.8 mm/yr incremental slip rates, respectively (Mozafari et al., 2021). Average slip rate is calculated to be 1.9 mm/yr. The historical earthquakes of 17 AD struck the Manisa region, and most probably caused displacement of the Manastır fault scarp (Mozafari et al., 2021).

A total of 44 samples were collected from a ca. 2.7 m high sampling profile along the 4 m high Mugırtepe fault surface (Akçar et al., 2012). A seismic event at ca. 6.5 ka with a minimum slip amount of ca. 2.7 m and a vertical slip rate of greater than 0.3 mm/yr was identified (Mozafari et al., 2021). Since only one earthquake has been modeled the incremental and average slip rates are identical and considered as the minimum.

### Büyük Menderes Graben

The approximately 140 km long Büyük Menderes Graben strikes E-W and continues with a change in strike to a NE-SW trend towards the Aegean Sea (Fig. 1). The graben is bounded by the Menderes Massif to the north and south and is filled with Quaternary sediments. Faults of varying scales define the horst-graben system, of which many extend along the northern edge of the entire structure, named the Büyük Menderes Fault Zone (Altunel et al., 2009; Emre et al., 2018; Sümer et al., 2013). The Priene-Sazlı fault (Mozafari, et al., 2019) and the Kalafat and Yavansu faults (Mozafari, et al., 2019) occur along this graben system, and have been studied to model their seismic history using fault scarp dating with cosmogenic ^36^Cl (Fig. 1).

The 15 km long Kalafat fault is a WNW-trending normal fault with a minor component of right-lateral movement on the northern side of Kalafat Mountain, SE of Kuşadası City (Fig. 1). The fault juxtaposes the footwall of Cycladic Massif bedrock against the hangingwall of Miocene volcano-sedimentary rocks overlain by colluvium. There is no seismic record attributed to the Kalafat fault. A total of 54 samples were collected from ca. 4.6 m of the fault surface along two parallel sampling strips (Mozafari, et al., 2019). Analysis of these samples indicates that three seismic events occurred at ca. 15, 8, and 4 ka, with dip-slip components of ca. 0.7, 1, and 3 m, respectively (Mozafari, et al., 2019). The incremental slip rates from the oldest to youngest modeled events are re-estimated to be greater than 0.1, 0.2, and 0.9 mm/yr, respectively, while the average slip rate is 0.3 mm/yr.

The Yavansu fault is generally an E-W trending normal fault with a minor component of dextral movement in the southern slope of Kalafat Mountain. The footwall bedrock is composed of Cycladic Massif, and is juxtaposed against Quaternary sediments on the hangingwall. Estimate of the fault length along the strike direction is 25 km with the possibility of extension of additional 25 km in the sea (Hancock & Barka, 1987). There are records of several earthquakes in Kuşadası during 1751**–**1893 AD (Soysal et al., 1981). The majority of the seismic events reported in the western extension of the Yavansu fault occurred north of Samos Island (Fig. 1), but none are directly assigned to activity of the Yavansu fault. To model the paleoseismicity of this fault, a total of 68 samples were collected from ca. 6.6 m along two sampling strips (Mozafari, et al., 2019). The best fit results for the Yavansu fault modeled ages for seismic events of ca. 8, 4, and 2 ka, with dip-slip component of associated displacement of ca. 0.6, 3.5, and 2.6 m, respectively. The average values of the incremental slip rates were reevaluated to be greater than 0.1, 1.7, and 0.9 mm/yr for the seismic events from oldest to youngest, while the average slip rate was ca. 0.6 mm/yr.

The Priene-Sazlı fault extends along the margin of the westernmost heights of the Büyük Menderes Graben (Fig. 1). The length of the fault is estimated to be in the range of 30 to 40 km (Altunel et al., 2009; Duman et al., 2011; Emre et al., 2013; Şaroğlu et al., 1992; Sümer et al., 2013*).* Generally, the Cycladic Massif constitutes the bedrock of the footwall, while the hangingwall is mantled by Quaternary sediments of the graben basin. The youngest fault plane solution indicates normal faulting with a minor component of right-lateral movement. Two major earthquakes related to the Priene-Sazlı fault have been recorded. A historical earthquake occurred in 68 AD with an intensity of VII at the NE termination of the fault (Ergin et al., 1967). The instrumental earthquake of Söke-Balat occurred on July 16, 1955 with a magnitude of 6.8 and hit the region causing heavy damage (Öcal, 1958; Şengör, 1987). A total of 117 samples were collected from the ca. 12 m high fault scarp along four horizontally offset sampling strips (Mozafari, et al., 2019)*.* Four seismic events were modeled at ca. 8, 6, 4, and 2 ka with the dip-slip components of associated displacement of ca. 3.4, 1.5, 1.4, and 1.5 m, respectively (Mozafari, et al., 2019). The modeled earthquake of ca. 2 ka is compatible with the historical earthquake of 68 AD. The mean incremental slip rates were re-estimated to be greater than 1.3, 0.5, 0.7, and 0.5 mm/yr, respectively, with an average vertical slip rate of 0.8 mm/yr.

## Study areas

The E-W-trending Rahmiye fault is at least 7 km long, and is a south-dipping, dextral dip-slip fault situated in the vicinity of Rahmiye village at the northwestern end of the Gediz-Alaşehir Graben (Figs. 1, 2, 3 and Additional file 1: Fig. S2). The fault is projected to be the continuation of the 40 km long NNW-SSE trending Akselendi fault, which is known as one of the boundary faults of the Gediz-Alaşehir Graben. The western two thirds section of the fault is characterized by a geomorphologic relief of over 300 m, while this value is lower by a factor of two in the eastern section. At the section of the Rahmiye fault that was sampled, the footwall of the fault is made up of late Jurassic-Cretaceous limestone juxtaposed against the Quaternary sediments of the hangingwall (Fig. 2). The largest known instrumental earthquakes in the area surrounding the Rahmiye fault were recorded on September 9, 2016 Mw = 5 and January 22, 2020 Mw = 5.6 approximately 3 km NW and 20 km N of the sampling site, respectively (Kandilli Observatory and Earthquake Research Institute, Boğaziçi University).

The Ören fault belongs to the Gökova Graben system, which is an E-W trending, asymmetric structure with a length of ca. 120 km (Görür et al., 1995), in southwest Anatolia (Fig. 1). The northern margin of the graben with a height of more than 1000 m, is composed of Lycian Nappes, which also constitute the basement of this region. The geomorphological relief in the eastern half is two times higher than the western half of the graben (Tur et al., 2015). This margin is controlled by the approximately 100 km long Gökova Fault Zone, which consists of several 55°–65° south-dipping normal fault segments, such as the Ören fault. Well preserved carbonate fault scarps of the Lycian Nappes contain kinematic indicators, namely fault striae and grooves, indicating a dip-slip normal character of the fault. The societal impact and importance of a seismic study in this segment of the fault is certainly related to the Kemerköy thermal power plant, which is constructed along the fault strike. Our sampling site is situated ca. 2-km east of this huge infrastructure. At the sampling site, in the center of the Gökova Fault Zone strike, the Lycian nappes and Tauride tectonic units constitute the bedrock of the scarp as footwall juxtaposed against the Quaternary alluvium as hangingwall (Fig. 4). According to the historical earthquake catalogues, many destructive earthquakes were recorded around the Gökova Gulf between 222 BC and 1896 AD (Ambraseys & White, 1997; Guidoboni et al., 1994; Yolsal et al., 2007). On July 20, 2017, a destructive earthquake occurred in the western part of the Gökova Graben, 18 km south of Bodrum City (Fig. 1). The results indicate that an approximately 65 km long fault section was activated during the mainshock of the 2017 Bodrum-Kos Earthquake (Tiryakioğlu et al., 2018). In addition, Papadopoulos et al. (2019) assert that a south-dipping normal fault at the western end of the Gökova Fault Zone is responsible for this E-W oriented normal faulting event. However, in the eastern and central parts of the graben, the closest earthquakes to our sampling site hit the area on May 23, 1941 (Mw: 5.4), December 13, 1941 (Mw: 6.3), April 25, 1959 (Mw: 5.9), August 04, 2004 (Mw: 5.3), and December 20, 2004 (Mw: 5.3) (Tiryakioğlu et al., 2018, and references therein), and also on January 10, 2005 (Mw: 5.5) (United States Geological Survey National Earthquake Information Center) (Fig. 4).

## Methodology

### Fieldwork

Fieldwork was performed over several weeks during the summer of 2014. Geology maps with scales of 1:100,000, topographic maps with scales of 1:25,000, active fault maps of Turkey with scales of 1:250,000 and 1:1,000,000 (Duman et al., 2011; Emre et al., 2013; Şaroğlu et al., 1992) were used as the database for the mapping. Maps were prepared using the ArcGIS 10 software. A digital elevation model (DEM) in different resolutions was produced to help with the preliminary selection of sampling sites, where needed.

### Sampling

We followed the guidelines proposed by Tikhomirov (2014), after Mitchell et al. (2001), for sampling. The least amount of weathering and erosion is essential for obtaining the most accurate results. To reconstruct at least one seismic event, a minimum of two meters of continuous sampling along the fault scarp from the ground level is advised (Schlagenhauf et al., 2010; Tikhomirov, 2014). An ideal sampling profile is a continuous strip parallel to the vertical component of slip. A sequence of profiles offset horizontally represents a practical alternative, where the fault surface is locally weathered (e.g., Akçar et al., 2012; Benedetti et al., 2002; Schlagenhauf et al., 2011). We examined a number of scarp outcrops along the Rahmiye fault in the Gediz-Alaşehir Graben and Ören fault along the Gökova Graben to identify the most suitable sites for our sampling. Based on our field geomorphological observations, we exclude any effect of surface weathering and erosion on the selected fault scarps (Mitchell et al., 2001). We also conclude that these scarps are purely exposed by tectonic slips and exclude any possibility of fault scarp exhumation by any erosional process (e.g., hill-slope movement, gravitational creep, and landslide). Both sites are located far away from any relay zones, river channels and structural boundaries (e.g., Cowie et al., 2017).

To access the higher part of the fault scarp and facilitate sampling, a ladder and/or scaffold is installed (Additional file 1: Fig. S3). First, the 15 cm wide sampling strip is marked along the fault surface upward, and parallel to the vertical component of slip. Second, the 8 to 10 cm high slabs are separated using markers from top to base along the fault surface (Additional file 1: Fig. S4a–b). Third, the samples are collected using a hand-held diamond saw, chisel and hammer (Additional file 1: Fig. S4c–d). In addition, the ground line, as the reference point for modeling, determines the previously covered section of the fault, which was exposed immediately by the most recent rupture. Possible recent removal of colluvium by human and its impacts were carefully considered in both sites and were taken into account for modeling. We identified the ground level based on a clear field evidence, which showed that some material from the top surface of the colluvium was recently removed. Though, the general dip of colluvium surface downslope was considered to define the exact level of colluvium after the most recent earthquake and before any probable recent colluvium removal. To double check the accuracy of the ground level based on the difference in the weathering degree of the surface, namely its freshness, we extrapolated the general colluvium dip as a hypothetical line and intersected with the fault surface. Both approaches revealed the same level for the ground line (Additional file 1: Fig. S5a). We preferred to collect subsurface samples; however, we were not permitted to dig a trench by the local authorities during the fieldwork; therefore, we were no deeper samples below the current ground level are collected.

Since the fault scarp geometry is essential for modeling the seismic history (e.g., Schlagenhauf et al., 2010; Tikhomirov, 2014), the following parameters are measured as precisely as possible in the field: (1) scarp dip (the angle between the fault scarp surface and a horizontal plane); (2) scarp height (length of the fault scarp surface from the ground level to the top of the scarp); (3) top surface dip (angle between the uppermost section of the fault scarp and a horizontal plane); and (4) colluvium dip i.e., the angle between the colluvium surface of about 15 m from the fault surface and a horizontal plane. During the fieldwork, the precise positions of all samples were recorded in reference to the ground line. Topographic shielding was determined as any barrier surrounding the scarps such as mountains, hills, ridges, and the scarps above. Furthermore, the colluvium density was calculated using a bucket of known volume and a balance in the field. Water content of bedrock and colluvium were used to be 0.2 and 1%, respectively.

In the Rahmiye fault (Gediz-Alaşehir Graben), a total of 87 samples were collected from the scarp along a ca. 6.5 m high sampling profile (Fig. 3b and Additional file 1: Fig. S3). The ground line was identified to be roughly 25 cm higher than today’s ground level (G, Fig. 3c). We attribute this to the extensive olive gardening in the region. Though, the first three slabs taken from the base of the fault were considered to be underground samples, i.e. samples shielded by the colluvium. In the Ören fault (Gökova Graben), we sampled the scarp outcrop along a ca. 12 m high strip (Figs. 4, 5 and Additional file 1: Fig. S3). A total of 127 samples were collected, of which six were assumed to be the samples from below the ground line, ca. 60 cm above the current ground level (Fig. 5a, b and Additional file 1: Fig. S6).

### Cosmogenic ^36^Cl Analysis

The samples were processed at the Surface Exposure Dating Laboratory of the University of Bern, following the protocol of Stone et al. (1996) and Ivy-Ochs et al., (2004, 2009), and the isotope dilution method described in Elmore et al. (1997) and Ivy-Ochs et al. (2004). Each sample slab was cut perpendicular to the surface, to reduce the thickness down to 2.5–3 cm. Afterward, the samples were crushed, ground, and sieved to obtain the 0.25–0.4 mm size fraction. Metal-shavings were removed from the sieved material using a hand-magnet. Then, the sieved material was leached overnight in 75 ml of 2 M HNO_3_, followed by four steps of rinsing with ultrapure water (18.2 MΩ.cm) to remove non-in situ Cl (Zreda et al., 1991). After rinsing, the leaching procedure was repeated one more time. Subsequently, the samples were dried on a hotplate at 60 °C. Aliquots of 12 g of sample from sample points at an interval of one meter along the fault scarp were analyzed with ICP and ICP-MS for major and trace elements at Actlabs Analytical Services, Canada. In addition, the Ca concentration of each sample was determined with ICP-MS. The samples were processed in batches of 15, including one full process blank. The samples were spiked with around 2.5 mg of ultra-pure ^35^Cl carrier in order to define the total Cl concentration (^35^Cl, ^37^Cl) (Ivy-Ochs et al., 2004, 2009), and were then dissolved in HNO_3_. The Cl concentration measurements are needed in order to calculate: (1) the ^36^Cl concentration in each sample; (2) the ^36^Cl production rate by low-energy neutron capture on ^35^Cl; and (3) the non-cosmogenic subsurface production of ^36^Cl. Afterward, following centrifuging, the impurities were separated from the samples. Approximately 10 ml of AgNO_3_ were added to the supernatant at 200 °C in the dark to precipitate AgCl. Then, the precipitated AgCl from each sample was collected and dissolved with 2 ml of NH_4_OH. To eliminate cations, the samples were then centrifuged. To avoid interference from the ^36^S isobar with ^36^Cl during Accelerator Mass Spectrometry (AMS) measurements, BaSO_4_ was precipitated by adding Ba(NO_3_)_2_ to the supernatant. Then, AgCl was re-precipitated. In the last stage, the solid AgCl decant was recovered and rinsed with ultrapure water and AgCl sample pills were pressed into Tantalum-lined copper targets for AMS measurements. The concentrations of total Cl and ^36^Cl were measured from a single target at the ETH AMS facility using an isotope dilution method (Christl et al., 2013; Ivy-Ochs et al., 2004; Synal et al., 1997). The stable ratio of ^37^Cl/^35^Cl was normalized to the neutral ratio ^37^Cl/^35^Cl = 31.98% of the K382/4 N standard and the machine blank. Ratios of ^36^Cl/^35^Cl derived from the measurements were normalized to the ETH internal standard K382/4 N with a ^36^Cl/Cl value of (17.36 ± 0.35) × 10^–12^ (Christl et al., 2013). The Surface Exposure Dating Laboratory of the University of Bern and AMS facility at ETH Zurich participated in the CoCal-N intercomparison study (Mechernich et al., 2019). The measured ^36^Cl and total natural Cl concentrations agreed well with the values from other laboratories. The sulfur correction to the measured ^36^Cl/^35^Cl ratio was negligible. The measured ^36^Cl/^35^Cl ratios of the samples were also corrected for a full process blank ratio of (1 ± 0.02) × 10^–15^; however, the contribution of the blank correction to the measured ratios was less than 1%.

### Fault scarp dating

During the last 20 years, fault scarp dating using in situ produced ^10^Be, ^26^Al, ^36^Cl, and ^14^C has been used to reconstruct the age of past ruptures of normal faults. ^36^Cl is used to date normal faults developed in carbonate rocks, as pioneered by Zreda and Noller (1998) on the Hebgen Lake fault scarp, USA. Since then, the seismic activity of carbonate fault scarps has been reconstructed using cosmogenic ^36^Cl at various locations in the eastern Mediterranean and the Middle East (Akçar et al., 2012; Beck et al., 2018; Benedetti et al., 2002, 2003, 2013; Carcaillet et al., 2008; Cowie et al., 2017; Goodall et al., 2021; Iezzi et al., 2021; Mechernich et al., 2018; Mitchell et al., 2001; Mouslopoulou et al., 2014; Mozafari et al., 2019, 2019, 2021; Palumbo et al., 2004; Schlagenhauf et al., 2010, 2011; Tesson & Benedetti, 2019; Tesson et al., 2016, 2020; Tikhomirov et al., 2014, 2019). Indeed, analysis of ^36^Cl profile along the fault scarp surface is an intricate challenge that requires a modelling code to translate isotope concentrations into ages and dip-slips. Several research groups provided such open access codes (Beck et al., 2018; Cowie et al., 2017; Goodall et al., 2021; Schlagenhauf et al., 2010; Tesson & Benedetti, 2019; Tikhomirov et al., 2019). These codes employ the same parameters and production rate constants, but they use different approaches to statistical matching of the measured ^36^Cl data. A detailed investigation and comparison of results from data sets using existing codes would be interesting topic to explore, but it is beyond the scope of the present study. Therefore, we applied—Fault Scarp Dating Tool (FSDT)—(Tikhomirov et al., 2019) to calculate the accumulation of cosmogenic ^36^Cl and reconstruct the paleoearthquake history of Rahmiye and Ören fault scarps in western Anatolia in order to provide a consistent comparison of our results with the recently published data (Mozafari et al., 2019, 2019, 2021). The FSDT code and input files used for the analysis of the scarps are provided as Additional file 3 that allows for result reproduction and analysis transparency.

We use Sea Level High Latitude (SLHL) production rates, attenuation lengths and scaling scheme (Table 1) to legitimately compare rupture history of seven fault scarps and present an overview of seismic activity in a vast area of western Anatolia. The code takes into account all production reactions of in-situ ^36^Cl, including spallation and capture by neutrons as well as muon-induced reactions (Liu et al., 1994; Phillips et al., 1996, 2001; Stone et al., 1996, 1998; Alfimov & Ivy-Ochs, 2009; Schimmelpfennig et al., 2009). Rates of production of cosmogenic ^36^Cl by high-energy neutron spallation on Ca (Stone et al., 1996), on K (Evans et al., 1997), on Ti (Fink et al., 2000), and on Fe (Stone, 2005) were given in Table 1. Furthermore, the production rate of epithermal neutrons from fast neutrons in the atmosphere at the land/atmosphere interface was calculated based on Alfimov and Ivy-Ochs (2009). In addition, to scale to the local production rates, we applied the scaling factors of Stone (2000) attributed for a constant geomagnetic field.

**Table 1:** Input parameters of the sampled fault scarps

	Rahmiye fault	Ören fault
Latitude Longitude Altitude	38.86372° N 27.80148° E 89 m	37.04088° N 27.92949° E 11 m
Scarp strike Scarp dip	N82°E 64°	N73°E 58°
Colluvium dip Scarp height Top surface dip	10° 6.4 m 22°	10° 12.2 m 34°
Rock density Colluvium density Rock water content Colluvium water content	2.4 g/cm^3^ 1.7 g/cm^3^ 0.2% 1%	2.4 g/cm^3^ 1.7 g/cm^3^ 0.2% 1%

Spallation on Ca of 48.8 ± 3.5 at g^−1^ yr^−1^ (Stone et al., 1996)

Spallation on K of 170 ± 25 at g^−1^ yr^−1^ (Evans et al., 1997)

Spallation on Ti of 13 ± 3 at g^−1^ yr^−1^ (Fink et al., 2000)

Spallation on Fe of 1.9 ± 0.2 at g^−1^ yr^−1^ (Stone, 2005)

Epithermal neutrons from fast neutrons: 760 ± 150 n/g^−1^ yr^−1^ (Alfimov & Ivy-Ochs, 2009)

High-energy neutron attenuation length: 208 g cm^−2^ (Gosse & Phillips, 2001)

Muonic production following Heisinger et al., (2002a, 2002b)

Scaling scheme for a constant geomagnetic field: (Stone, 2000)

Model rupture histories with fixed number of seismic events is generated with Monte-Carlo method in a constrained solution space of the modeling parameters: age of ruptures, dip-slip displacement related to each rupture, erosion rate of the fault surface, and the beginning of exposure. “Beginning of exposure” accounts for inherited ^36^Cl and refers to the start of the ^36^Cl build-up in the fault scarp at depth, which is calculated 25% uncertainty within 2σ. Therefore, this reported age does not indicate any exposure and any earthquake event. Though, at the beginning of cosmogenic ^36^Cl accumulation, the sampling surface is assumed to be at depth, not displaced yet and covered by colluvium, hypothetically this depth is possibly up to several meters. In this study, we use the term “beginning of ^36^Cl accumulation” to avoid any misunderstanding.

Among a large number of tested scenarios, in terms of number of earthquakes, age of earthquakes and displacement value, the one with the lowest statistical criteria, including chi-square (Χ^2^), weighted root mean square (RMSw), and Akaike information criterion (AICc), is accepted as the most probable solution. The model takes into account the sources of uncertainties that may influence the modeled age and slip value, including ^36^Cl and parent element concentration, production rates, and scarp geometry. In order to include any inaccuracy caused by the identification of the ground line by extrapolating the colluvium surface line along the cross-section perpendicular to the sampling site, we set an uncertainty of usually 20 cm to search for the most realistic ground level in the modeling. Overall, these errors cause an upper estimate of the modeled age and slip of 25% and 15% uncertainty within 2σ, respectively (Tikhomirov et al., 2019). The older the modeled age and slip are, the higher the uncertainty. The number of events modeled by FSDT is the minimum, since the code only explores major earthquakes with the potential of fault displacement. Furthermore, distinguishing between multiple earthquakes that occurred within the uncertainty ranges for the age and slip is unlikely. Accordingly, one modeled seismic event can represent either a single earthquake or clustering earthquakes, caused by several ruptures within short time intervals.

### Seismic capacity

No seismological technique is able to explicitly predict the magnitudes of future earthquakes. However, the probable magnitude can be approximated based on empirical relationships, as a logarithmic parameter that links the fault surface length (SRL) to the magnitude of the earthquake. In this study, we used the approaches of Pavlides and Caputo (2004) and Wells and Coppersmith (1994), based on normal faults in the Aegean region and worldwide, respectively, regardless of modeling (Eqs. 1 and 3 in Additional file 2: Table A). Once we calculated the potential earthquake magnitude, the probable maximum vertical displacement (MVD) values, i.e. slip amounts, were determined using the same approaches (Eqs. 2 and 4 in Additional file 1: Table S1). Afterward, we computed the magnitude an occurring earthquake may have based on the modeled slips (Eqs. 5 and 6 in Additional file 2: Table S1) and evaluated the plausibility by comparing the modeled with the theoretical magnitude and slip values.

## Results

Detailed mapping showed the nature of the Rahmiye fault as an escarpment composed of successive scarps of varying levels. At least three levels of scarps can be traced spaced a couple of meters along almost the entire strike of the fault, of which the lowermost scarp surface is considered to be active and characterized by clear triangular facets (Additional file 1: Fig. S7), an obvious indicator of a normal fault activity. The Rahmiye Fault seems to act as the southern boundary of a group of inactive normal and strike-slip faults of older generations with general N-S and NW–SE trends, respectively, while the fault strike intersects the latter (Fig. 2). The lower section of the escarpment with a dip varying between 48°–69° along the fault strike is covered by 10°–20° dipping colluvium in the lowermost part extending towards the Gölmarmara Basin (Additional file 1: Fig. S5). The fault surface is easily traceable along its strike; however, selected surface for the sampling has the longest height along the entire fault length. The continuous nature of the fault along its strike along with easily observable constant colluvium dip assures that there is no significant erosion and deposition on both footwall and hangingwall (Additional file 1: Figs. S2 and S5). Traces of old excavations, probably conducted by ancient Romans exist on the fault surface, just to the right of the sampling profile (Fig. 3a, b and Additional file 1: Fig. S2). Two different patterns of weathering, which points to a stepwise exhumation are visible on the fault surface. The upper half of the fault surface covering ca. 3 m is highly fractured both horizontally and vertically, while in the lower part strong fracturing is missing. In addition, the dissolution marks have diverse orientations on the upper and lower surfaces (Fig. 3a and Fig. S8). Corrugations are well preserved on the lower part of the fault surface indicating dextral dip-slip movement of the fault. This is an additional evidence for negligible weathering and erosion on the fault surface (Additional file 1: Fig. S8). The scarp, colluvium, and top surface dips were measured to be 64°, 10° and 22°, respectively (Fig. 3c).

Similarly, in the sampling surface of the Ören Fault, at least three levels of scarps, in addition to that of the lower most targeted for sampling, are obvious (Fig. 5a). A geomorphological relief of over 300 m including retreated scarps is observed in the sampling site. Insignificant weathering and erosion are also evident by the fresh dip-slip corrugations indicating the pure normal character of the fault (Additional file 1: Fig. S6). The scarp, colluvium, and top surface dips were measured to be 58°, 10° and 34°, respectively (Fig. 5b). No differentiation of the weathering pattern was observed on the fault surface.

### Cosmogenic ^36^Cl analysis

The position of the samples along the vertical transects, sample thickness, cosmogenic ^36^Cl concentration, natural Cl concentration, and their uncertainties, and the Ca concentration of the samples from the Rahmiye and Ören fault scarps are given in Additional file 2: Tables S2 and S3. In Additional file 2: Table S4, the blank measurements associated with each sample batch are listed.

**Fig. 6:**
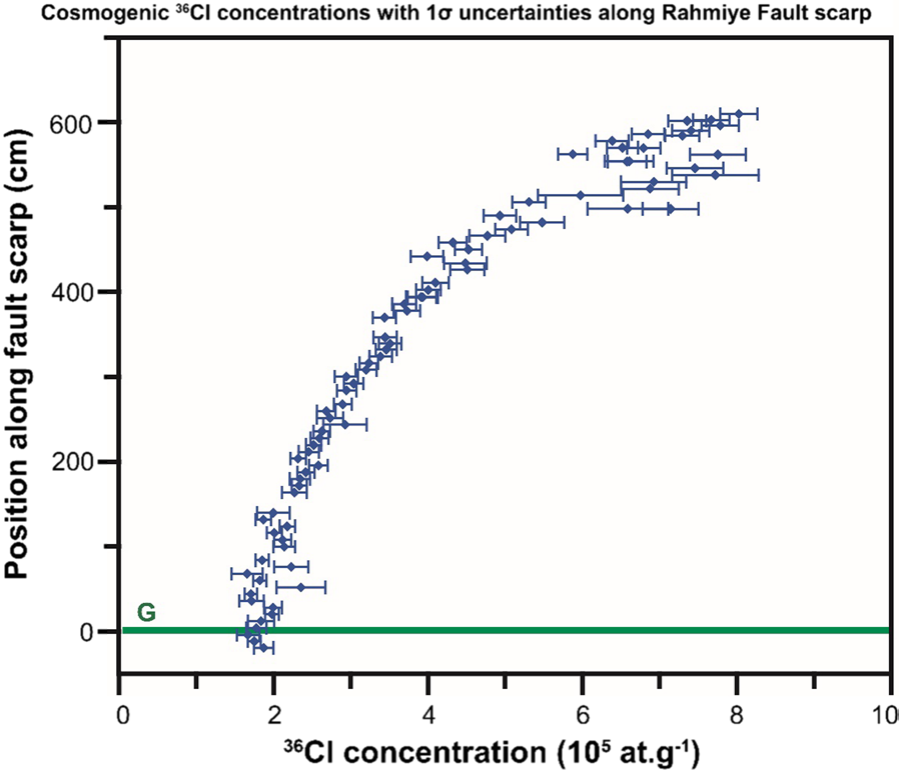
Cosmogenic ^36^Cl concentrations with 1σ uncertainties versus height along the Rahmiye fault scarp surface. G marks the ground line

**Fig. 7:**
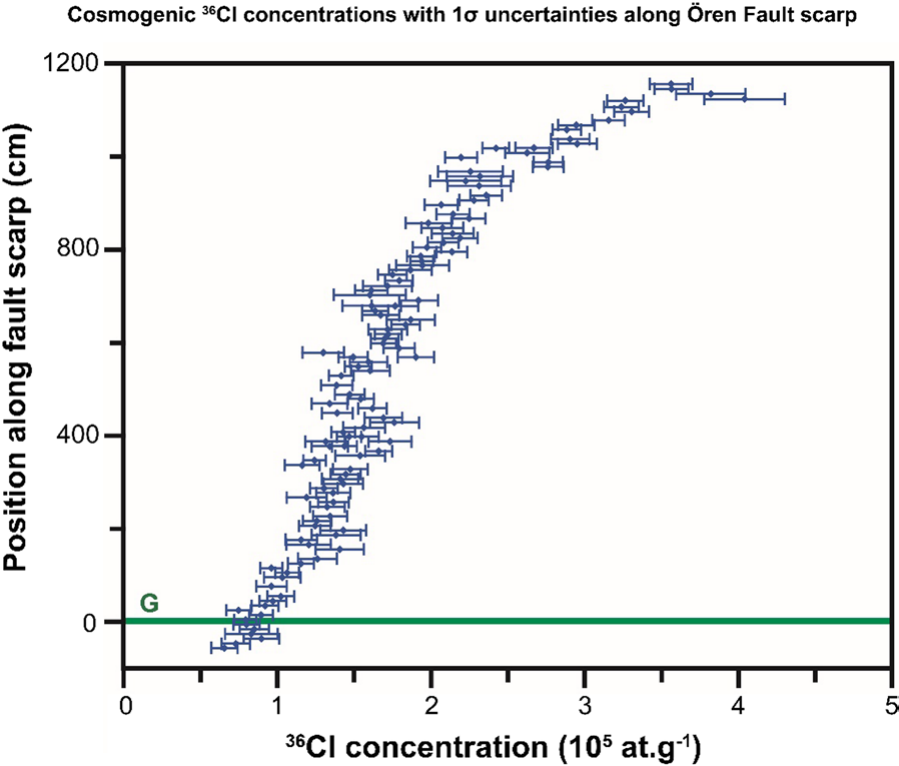
Cosmogenic ^36^Cl concentrations with 1σ uncertainties versus height along the Ören Fault scarp surface. G marks the ground line

The major and trace elements were measured in nine and 13 representative samples from the Rahmiye and Ören fault scarps, respectively, and their average values used for modeling (Additional file 2: Tables S5 and S6). The fault scarp parameters and default rates of the ^36^Cl production are given in Table 1. The density of the colluvium at both faults was measured to be 1.7 g/cm^3^ in the field, and the density of the limestone was 2.4 g/cm^3^. Measured cosmogenic ^36^Cl concentrations with their associated uncertainties were plotted versus sampling height for the Rahmiye and Ören faults, respectively (Figs. 6 and 7).

### Modeled seismic activity

Using FSDT we have modeled at least 20 rupture history scenarios with different number of seismic events, 3 to 5 for Rahmiye and 3 to 6 for the Ören fault. Each scenario was modeled and statistically tested more than 100,000 times with randomly generated modeling parameters applying Random Walk Monte-Carlo algorithm (Additional file 2: Table S7).

**Fig. 8:**
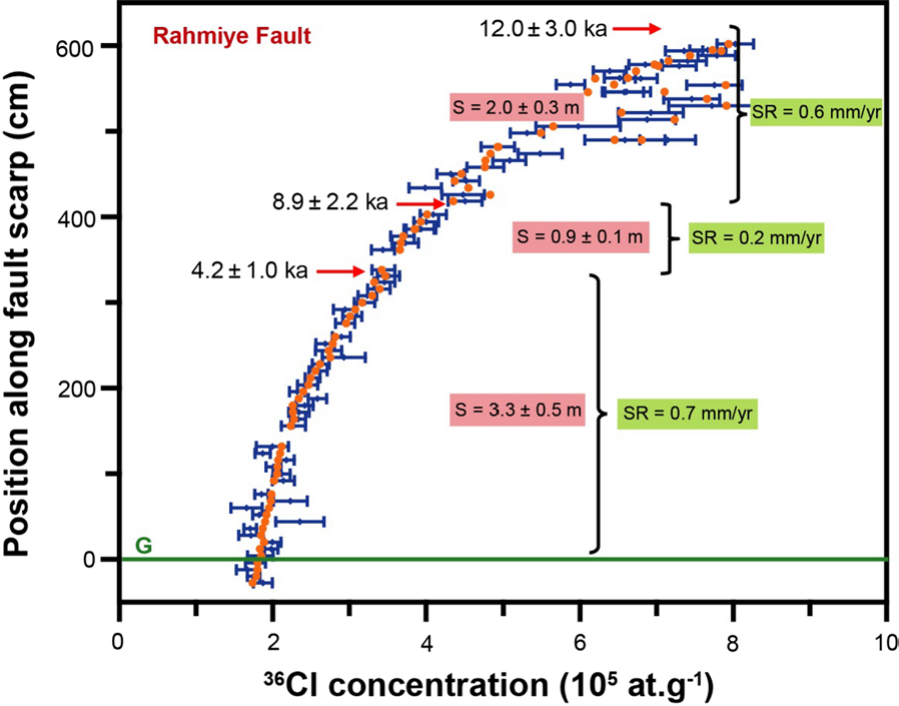
Best fit (filled circles) of the data for the samples from the Rahmiye fault scarp with a three rupture model. Black dots with 1σ uncertainties are measured ^36^Cl concentrations. The arrows mark the colluvium positions before the modeled seismic event. S defines the amount of slip. Short-term slip rates (SR) are calculated from the throw/maximum vertical displacement

A scenario of three seismic events for the Rahmiye fault was populated with 102,408 simulations and provided the best fit solution (Additional file 1: Fig. S9). The statistical parameters for this best scenario are Χ^2^ = 1.3, RMSw = 1.1 and AICc = 248 (Additional file 2: Table 2). The results showed the beginning of ^36^Cl accumulation about 47 ka, which was followed by three seismic events at 12.0 ± 3.0, 8.9 ± 2.2, and 4.2 ± 1.0 ka, with associated slip of 2.0 ± 0.3, 0.9 ± 0.1, and 3.3 ± 0.5 m, respectively (Fig. 8). The modeled seismic events on the Rahmiye fault display a relatively regular recurrence interval.

**Table 2:** Results for the data set from the Rahmiye and Ören fault scarps along with statistical criteria

Database	Event number	Beginning of ^36^Cl accumulation (ka)	Age (ka)	Slip (m)	Statistical criteria
Rahmiye	3	47	12.0 8.9 4.2	2.0 0.9 3.3	Χ^2^ = 1.3 RMSw = 1.1 AICc = 248
Ören	4	25	8.5 6.0 4.3 1.9	3.4 4.2 2.3 1.8	Χ^2^ = 1.4 RMSw = 1.1 AICc = 485

Chi-squared Value (Χ^2^)

Weighted root mean square (RMSw)

Akaike information criterion (AICc)

**Fig. 9:**
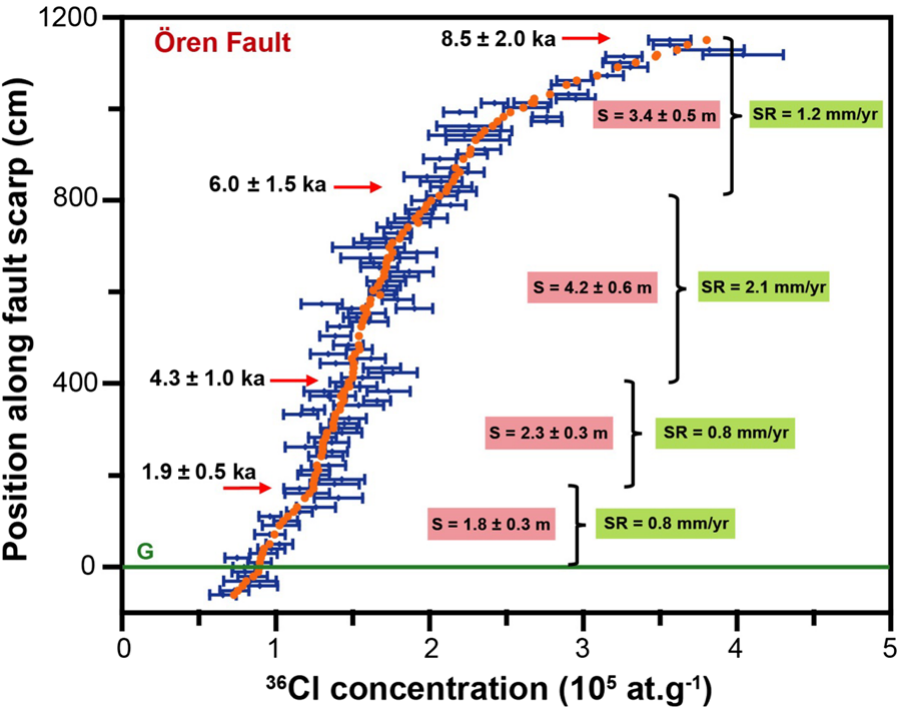
Best fit (filled circles) of the Ören fault scarp dataset with a four rupture model. Black dots with 1σ uncertainties are measured ^36^Cl concentrations. The arrows mark the colluvium positions before the modeled seismic events. S defines the amount of slip. Short-term slip rates (SR) are calculated from the throw/maximum vertical displacement

A scenario of four seismic events gave the best fit for the Ören fault with 107,428 simulations (Additional file 1: Fig. S9) and yielded statistical parameters of Χ^2^ = 1.4, RMSw = 1.1, and AICc = 485 (Table 2). The beginning of ^36^Cl accumulation is identified to be about 25 ka and the ages of the seismic events are 8.5 ± 2.0, 6.0 ± 1.5, 4.3 ± 1.0, and 1.9 ± 0.5 ka, with vertical components of associated slip of 3.4 ± 0.5, 4.2 ± 0.6, 2.3 ± 0.3, and 1.8 ± 0.3 m, respectively (Fig. 9). Based on these results, a regular recurrence interval of approximately 2000 years for earthquakes of the Ören fault was calculated.

### Slip Rates of the Rahmiye and Ören Faults

**Fig. 10:**
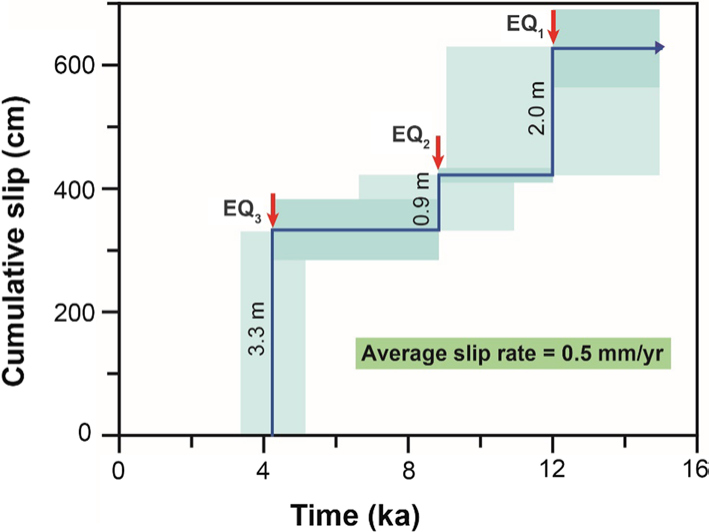
Cumulative slip versus time with uncertainties of seismic events ages and colluvium level obtained from modeling of the Rahmiye fault dataset. The long-term slip rate is 0.5 mm/yr

Incremental slip rates are calculated by dividing throw/maximum vertical displacement by the corresponding duration between two successive earthquakes or earthquake clusters (e.g., Benedetti et al., 2002; Onderdonk et al., 2015; Ren et al., 2013; Tsodoulos et al., 2016). The incremental slip rates, in order of the oldest to youngest reconstructed ruptures, are estimated to be 0.6, 0.2, and 0.7 mm/yr for the Rahmiye fault (Fig. 8). It should be noted that for all faults modeled using FSDT, the oldest incremental slip rate is considered to be the lower bound. The average slip rate is estimated by dividing the accumulated throw that occurred in the time window of the oldest modeled earthquake and the current time. The calculated average slip rate is 0.5 mm/yr (Fig. 10).

**Fig. 11:**
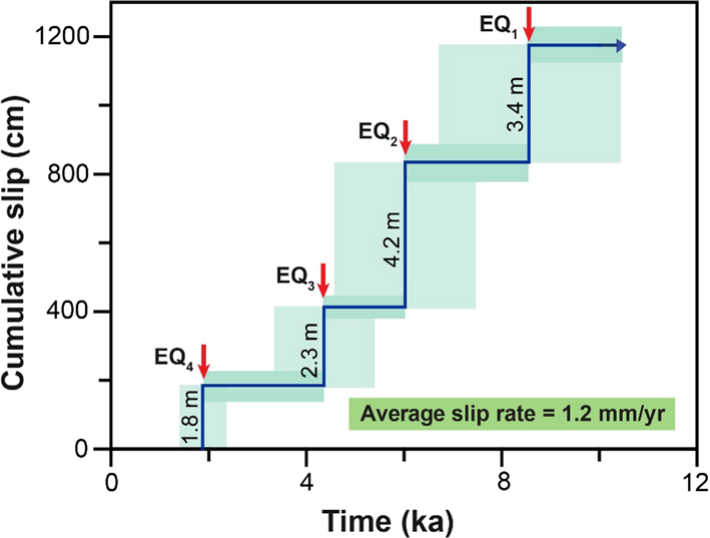
Cumulative slip versus time with uncertainties of seismic events ages and colluvium level obtained from modeling of the Ören fault dataset. The long-term slip rate is 1.2 mm/yr

The maximum vertical displacements between the ruptures of the Ören fault give an incremental slip rate of 1.2, 2.1, 0.8, and 0.8 mm/yr for the oldest to the youngest earthquakes, respectively (Fig. 9). The calculated average slip rate is 1.2 mm/yr (Fig. 11), considering cumulative throw occurred in the time span between the oldest modeled earthquake and the current time.

### Seismic Capability of the Rahmiye and Ören Faults

Regardless of modeling, but based on its length of 7 km, the Rahmiye fault can produce an earthquake of magnitude 6.0–6.2 on average (Eqs. 1 and 3 in Table 3, respectively). However, the probable length of the fault of about 40 km, results in an earthquake with a potential magnitude of 6.9–7.0. To approximate the average vertical displacement, similar empirical approaches were used (Eqs. 2 and 4 in Table 3). A fault length of 7 km yields a maximum vertical displacement (MVD) and slip of a few centimeters, whereas these values increase to 1.3 or 2.0 m on average, respectively, if the length of the fault is 40 km. If the shorter length of the fault is considered, the sampled fault surface required a large number of earthquakes to be exposed on more than 6 m of sampling profile height. As stated above, FSDT cannot identify the small-scale slips within the uncertainty of modeling as separate events. Even if each of the three modeled seismic events are composed of series of smaller slips, they should have occurred in a closely timed window. We exclude this possibility because the occurrence of such repeated seismic activity e.g. over ten earthquakes in short time interval is not known across Anatolia, neither in instrumental nor in historical records. In addition, continuous pattern of dissolution marks at least in the lower part of the fault reveals that most probably this section exhumed at once or alternatively very close in time, when the fault surface was still smooth for the formation of the continuous rills. The field evidence regarding capacity of the fault to produce larger displacements in addition to calculation above suggests that the Rahmiye fault should be an extension of the Akselendi fault (Fig. 2).

**Table 3:** Regression of SRL (Surface Rupture Length), magnitude (Ms/M) and vertical displacement (MVD/MD) calculated for Rahmiye and Ören faults regardless of modeling

	SRL/FL	7 km	40 km	100 km
Sin (θ) = vertical displacement/slip		Rahmiye fault (θ = 64°)		Ören fault (θ = 58°)
Pavlides and Caputo (2004)	Ms = 0.9 × Log (SRL) + 5.48 (Eq. 1)	6.2	6.9	7.3
	Log (MVD) = 1.14 × Ms–7.82 (Eq. 2)	MVD = 0.2 Slip = 0.2	MVD = 1.2 Slip = 1.3	MVD = 3.0 Slip = 3.6
Wells and Coppersmith (1994)	M = 4.86 + 1.32 × log (SRL) (Eq. 3)	6.0	7.0	7.5
	Log (MD) =–5.90 + 0.89 × M (Eq. 4)	MD (Slip) = 0.3	MD (Slip) = 2.0	MD (Slip) = 6

The unit of slip, MVD and MD is in meters

MVD (maximum vertical displacement) is converted to slip or MD (maximum displacement) by applying fault surface dip (sin (θ) = maximum vertical displacement/slip)

For the longer Ören fault, larger earthquake magnitudes are expected. If the Ören fault, with a considered length of 100 km, ruptures entirely following a seismic event, the probable earthquake would theoretically have a magnitude of 7.3–7.5 on average (Eqs. 1 and 3 in Table 3, respectively). Such large magnitudes are capable of having ruptured the fault with slip amounts of 3.6 or 6.0 m on average (Eqs. 2 and 4 in Table 3, respectively). Please note that these quantifications are plausible only if the whole fault is active. However, in reality such a long fault composed of different segments may involve breakage with or without the neighboring segments that were displaced through occurrence of floating earthquakes.

## Plausibility of earthquake modeling

In the previous section, the possible earthquake magnitude and the potential displacement of the Rahmiye and Ören faults calculated based on the fault length were presented, independent of the results derived from FSDT modeling (Table 3). Here, the seismic activity and rupture history of the studied faults are discussed together with the estimated magnitude required to produce the modeled amount of slip (calculations in Table 4), and both are compared with the theoretical magnitude and slip that can be generated by these faults (calculations in Table 3). One should note that, there is no approach to identify smaller seismic events by any existing fault scarp dating codes. Thus, the number of modeled events provided below is minimum, accordingly the estimated magnitudes are supposed to be maximum value.

**Table 4:** Regression of magnitude (MS/M) and vertical displacement (MVD/MD) for the Rahmiye and Ören faults

Sin (θ) = vertical displacement /Slip		Event	Rahmiye fault (θ = 64°)			Event	Ören fault (θ = 58°)		
			Slip* (m)	MVD (m)	Ms		Slip* (m)	MVD (m)	Ms
Pavlides and Caputo (2004)	Ms = 0.59 × Log (MVD) + 6.75 (Eq. 5)		Lowest Χ^2^	Average	Average		Lowest Χ^2^	Average	Average
		1	2.0 ± 0.3	1.8 ± 0.2	6.9	1	3.4 ± 0.5	2.9 ± 0.4	7.0
		2	0.9 ± 0.1	0.8 ± 0.1	6.7	2	4.2 ± 0.6	3.6 ± 0.5	7.0
						3	2.3 ± 0.3	2.0 ± 0.3	6.9
		3	3.3 ± 0.5	3.0 ± 0.4	7.0				
						4	1.8 ± 0.3	1.5 ± 0.2	6.9

		Event	MD (= Slip*) (m)	M		Event	MD (= Slip*) (m)	M	
Wells and Coppersmith (1994)	M = 6.61 + 0.71 × log (MD) (Eq. 6)		Lowest Χ^2^	Average			Lowest Χ^2^	Average	
		1	2.0 ± 0.3	6.8		1	3.4 ± 0.5	7.0	
						2	4.2 ± 0.6	7.1	
		2	0.9 ± 0.1	6.6					
						3	2.3 ± 0.3	6.9	
		3	3.3 ± 0.5	7.0					
						4	1.8 ± 0.3	6.8	

The unit of slip, MVD and MD is in meters

Slip or MD (maximum displacement) is converted to MVD (maximum vertical displacement) by applying fault surface dip (sin (θ) = maximum vertical displacement/slip)

^*^Modeled by the code

**Fig. 12:**
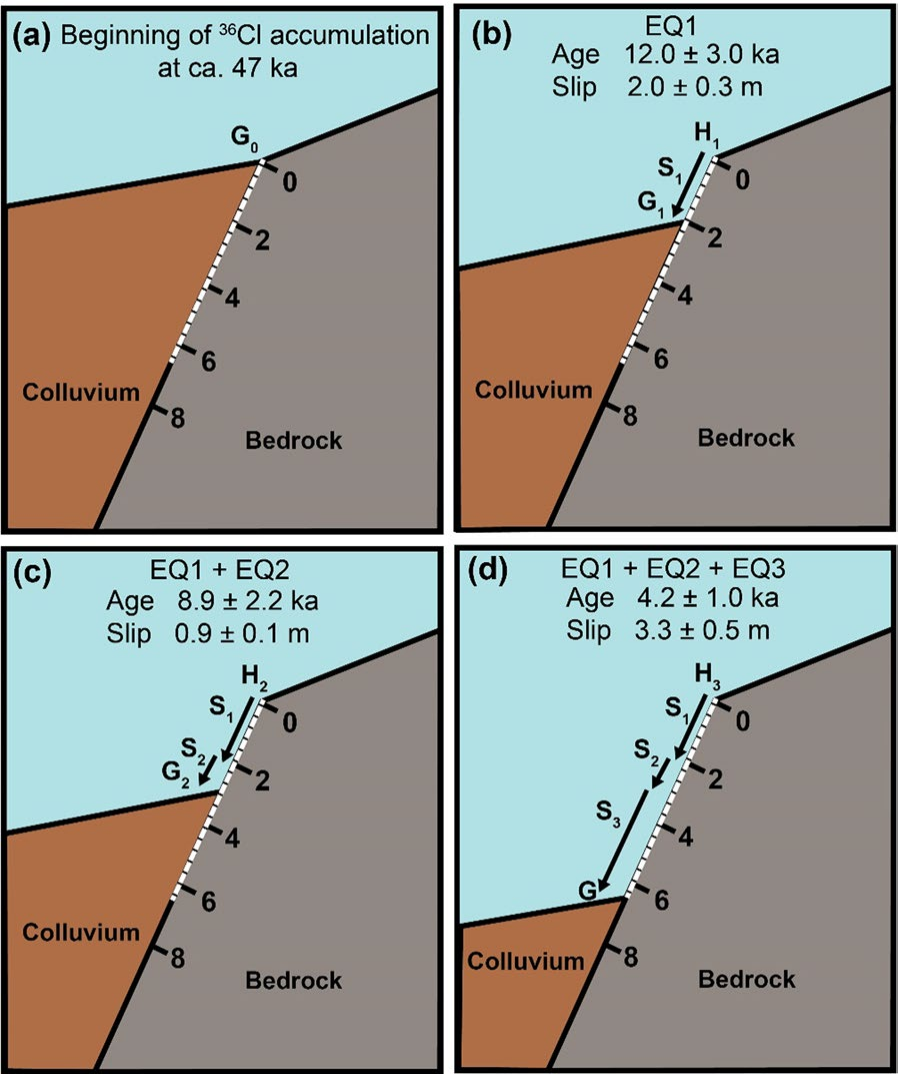
Cartoon of the Rahmiye fault ruptures (**a**) showing colluvium position before its activity period; **b**–**d** episodic fault exposure during three modeled seismic events. White dashed line shows the total sampled surface. The fault surface grade is in meters. H_0_ and G_0_ show scarp height and ground level prior to the first rupture. H_1_ to H_3_ indicate fault scarp height following earthquakes 1 to 3, respectively. G_0_ (ground level just before the first rupture); G_1_ and G_2_ are ground levels before the second and third ruptures, and G is the ground line. S_1_ to S_3_ represent the slip amounts of the three earthquakes

The summary of time-slip history of the Rahmiye fault is presented in Fig. 12. The modeled amount of slip produced by *Seismic Event 1* indicates the occurrence of one or two earthquakes with magnitudes of 6.8–6.9 (Eqs. 5 and 6 in Table 4). *Seismic Event 2* is interpreted to be resulted from a single earthquake of magnitude 6.6–6.7 (Eqs. 5 and 6 in Table 4). The associated slip produced by *Seismic Event 3,* the last rupture could be produced by two or three earthquakes of magnitude 7.0 (Eqs. 5 and 6 in Table 4). The model inference of a seismic event at this height on the scarp surface is supported by the field observation of an abrupt change in weathering features below this level (Fig. 3a). Moreover, the cross-cutting relationships of ancient excavation and vertical dissolution mark can be used as a simple but fascinating tool to obtain relative ages of earthquakes. The partial absence of dissolution mark crosscut by the middle excavation (Fig. 3a) reveals that the time, in which the exhumed fault surface was fresh enough for this runoff path to be formed, is older than the middle excavation. We interpret the excavations to be of Roman age based on the chiseling marks (cf. Yavuz, 2014), thus that part of the exhumed fault surface is older than 2 ka.

**Fig. 13:**
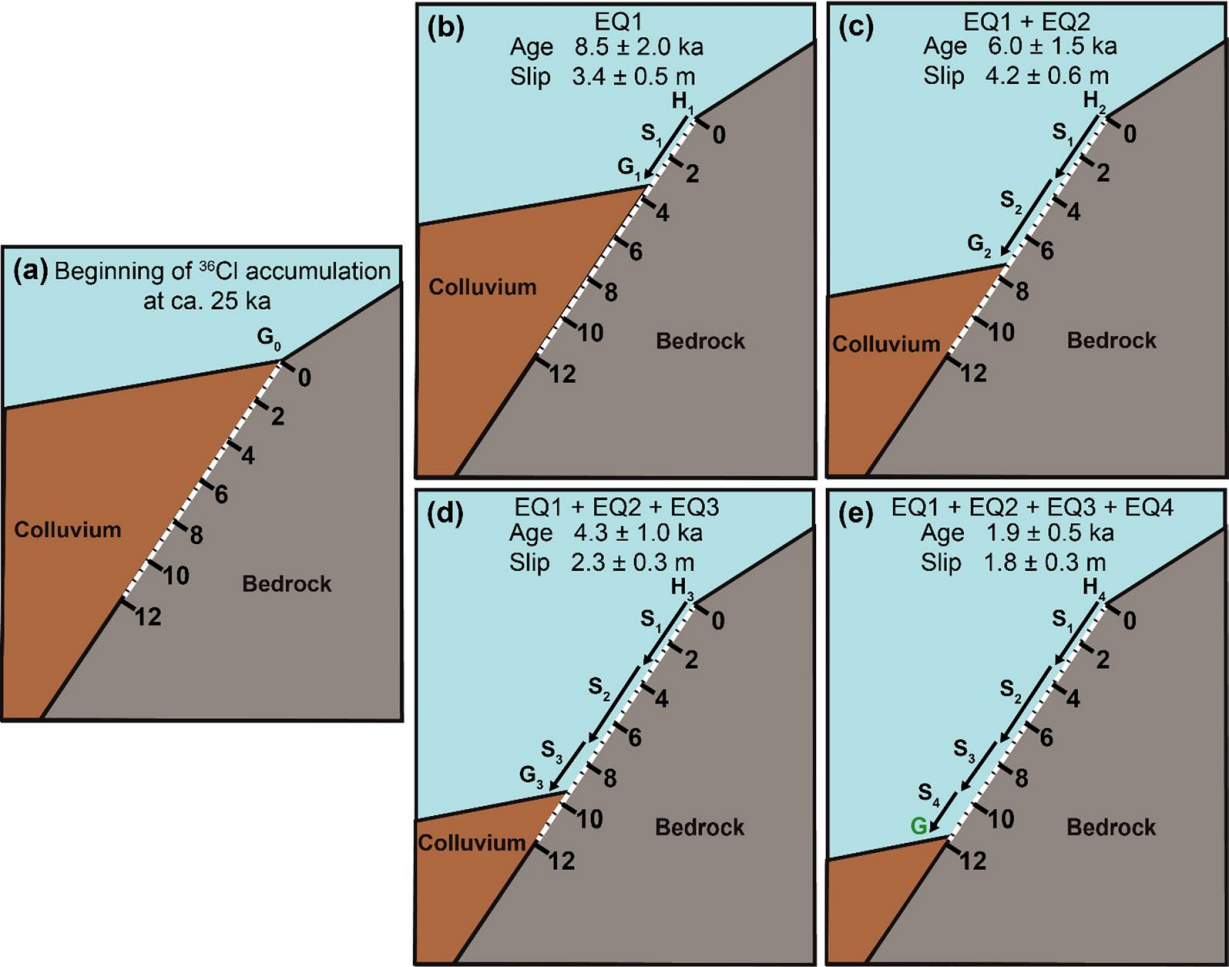
Cartoon of the Ören fault ruptures (**a**) showing colluvium position before its activity period; **b**–**e** episodic fault exposure during four modeled seismic events. White dashed line shows the total sampled surface. The fault surface grade is in meters. H_0_ and G_0_ show scarp height and ground level prior to the first rupture. H_1_ to H_4_ indicate fault scarp height following earthquakes 1 to 4, respectively. G_0_ (ground level just before the first rupture); G_1_ to G_3_ are ground levels before the second, third and fourth ruptures, and G is the ground line. S_1_ to S_4_ represent the slip amounts of the four earthquakes related to the past to recent events

The best fit results for the Ören fault database show the occurrence of four seismic events (Fig. 13). The corresponding slip by *Seismic Event 1,* indicates an earthquake of magnitude 7.0 (Eqs. 5 and 6 in Table 4). The associated slip of *Seismic Event 2* could be produced by a maximum of two earthquakes of magnitude 7.0–7.1 (Eqs. 5 and 6 in Table 4). *Seismic Event 3* could be resulted from an earthquake of magnitude 6.9 (Eqs. 5 and 6 in Table 4). The last rupture, *Seismic Event 4,* could be the result of an earthquake of magnitude 6.8–6.9 (Eqs. 5 and 6 in Table 4). According to these results, the modeling reveals that seismic events generated by the Rahmiye and Ören faults are not necessarily characterized by single earthquakes, but also clustering earthquakes that may have occurred as closely timed events.

## Discussion

In the following sections, we discuss the implications of our results, starting with the local scales of the individual studied faults followed by the main phases of activity at the scale of western Anatolia. Finally, we compare the calculated recurrence intervals and slip rates with the ones reported within the broader tectonic region of the eastern Mediterranean.

### Seismic activity in western Anatolia during the last 16 kyr

Modeling the seismic history of faults, especially estimating the recurrence interval, potential earthquake magnitude, and slip rate, is a key step in evaluating the associated seismic risks and helps to minimize the risks of damage to property and lives from future earthquakes. The importance of obtaining this information is even higher for densely populated urban areas like western Anatolia. Using cosmogenic ^36^Cl dating, the paleoseismic behavior of the Rahmiye fault (this study), the Manastır and Mugırtepe faults in the Gediz-Alaşehir Graben (Mozafari et al., 2021), the Kalafat, Yavansu and Priene-Sazlı faults in the Büyük Menderes Graben (Mozafari, et al., 2019; Mozafari, et al., 2019), and the Ören fault in the Gökova Graben (this study) in western Anatolia during the last 16 kyr were explored. Overall, a minimum of 20 seismic events have been identified showing that all the investigated faults experienced at least one rupture either as individual or clustered earthquakes through the Holocene. The results derived from the FSDT modeling of western Anatolia from this, and previous studies are summarized in Table 5. The faults are considered to be seismogenic faults, because they have the capacity to produce earthquakes of magnitude 5.0 or larger (McCalpin, 2009), which emphasizes the need for further earthquake geochronological studies. In light of the calculated average slip rates, slip per event, rupture length, magnitude of potential earthquakes, and recurrence intervals of the studied faults using ^36^Cl dating in western Anatolia, they are classified as low to moderately (class 3) active faults based on the classification of Cluff and Cluff (1984).

**Table 5:** Results for the data set from the examined fault scarps in western Anatolia

Graben/ Fault		Number of events	Beginning of ^36^Cl accumulation (ka)	Age (ka)	Slip per event (m)	Incremental slip rate (mm/yr)	Average slip rate (mm/yr)	Potential Magnitude based on fault length	Calculated magnitude based on modeled slip
Gediz-Alaşehir graben	Rahmiye	3	47	12.0 ± 3.0 8.9 ± 2.2 4.2 ± 1.0	2.0 ± 0.3 0.9 ± 0.1 3.3 ± 0.5	0.6 0.2 0.7	0.5	6.9–7.0	6.6–7.0
	Mugırtepe	1	27	6.5 ± 1.6	2.7 ± 0.4	> 0.3	0.3	6.9	6.9
	Manastır	2	9	3.5 ± 0.9 2.0 ± 0.5	3.3 ± 0.5 3.6 ± 0.5	> 2.2 1.8	1.9	6.9	7.0
Büyük Menderes graben	Kalafat	3	22	15.3 ± 3.8 8.4 ± 2.1 3.6 ± 0.9	0.7 ± 0.1 0.9 ± 0.1 3.1 ± 0.5	> 0.1 0.2 0.9	0.3	6.4–6.5	6.5–7.0
	Yavansu	3	12	7.9 ± 2.0 3.4 ± 0.8 2.0 ± 0.5	0.6 ± 0.1 3.5 ± 0.5 2.6 ± 0.4	> 0.1 1.7 0.9	0.6	6.7	6.5–7.0
	Priene-Sazlı	4	21	8.1 ± 2.0 6.0 ± 1.5 3.7 ± 0.9 2.2 ± 0.5	3.4 ± 0.5 1.5 ± 0.2 1.4 ± 0.2 1.5 ± 0.2	> 1.3 0.5 0.7 0.5	0.8	6.9	6.7–7.0
Gökova graben	Ören	4	25	8.5 ± 2.0 6.0 ± 1.5 4.3 ± 1.0 1.9 ± 0.5	3.4 ± 0.5 4.2 ± 0.6 2.3 ± 0.3 1.8 ± 0.3	1.2 2.1 0.8 0.8	1.2	7.3–7.5	6.8–7.1

**Fig. 14:**
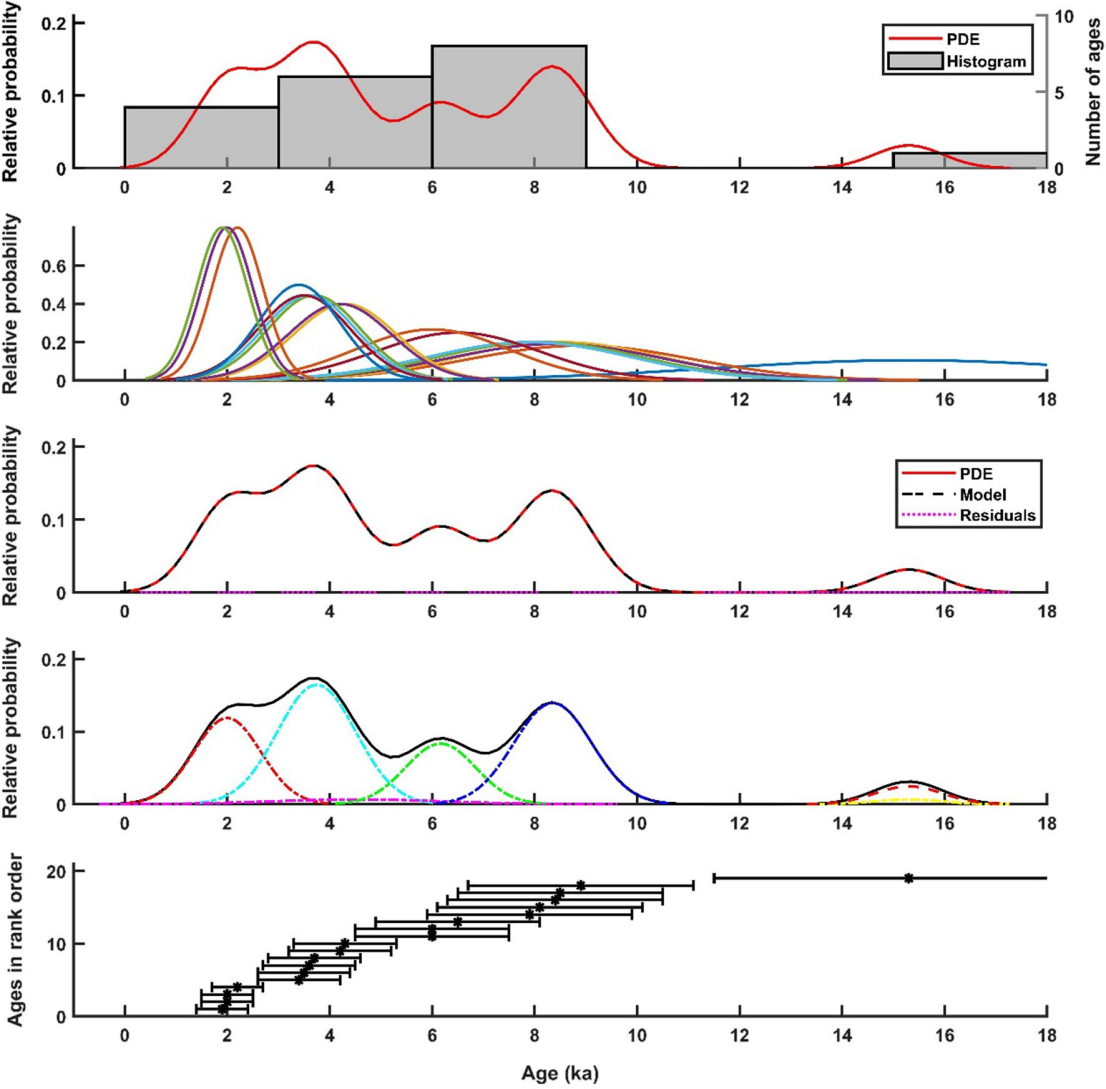
Comparison of modeled seismic events ages and their relative probability using Probabilistic Cosmogenic Age Analysis Tool (P-CAAT) (Dortch et al., 2021). Four main peaks imply the phases of activity in western Anatolia. Dots and error bars show the age of modeled seismic events with 2σ uncertainties

As mentioned above, knowledge about how the faults of a tectonic setting act during a long-time span in terms of occurrence as individual or clustering earthquakes is essential for earthquake hazard assessment. We used P-CAAT (Dortch et al., 2021) to plot and evaluate the reconstructed seismic events ages and the corresponding uncertainties. Based on mean value of modeled ages, showing four peaks and considering that the uncertainties of modeled ages are the upper bound, we conclude that the seismic activities in western Anatolia were evidently caused by clustering earthquakes of several faults with a recurrence interval of approximately 2000 years (Fig. 14). However, one should keep in mind that this estimation suffers from incompleteness of seismic records back in time and is merely an estimation mainly based on our fault scarp dating studies. In addition, stating whether and how these temporal correlations are spatially connected is not known. However, we classified the modeled seismic events into discrete groups based on time correlations, called “activity phases”. We consider the oldest seismic events of 15 and 12 ka, along the Kalafat and Rahmiye faults, respectively, to have been a local phase of activity; therefore, these events are not synchronized with the other modeled events. During *Activity Phase I* (ca. 8 ka), the oldest seismic events occurred on the Kalafat, Yavansu, Priene-Sazlı and Ören faults. The seismic event of ca. 8.9 ka on the Rahmiye fault is unlikely to be connected to this phase of activity. *Activity Phase II* (ca. 6 ka) is expressed in the Mugırtepe, Priene-Sazlı and Ören faults with a high distinction in terms of age correlation. In *Activity Phase III* (ca. 4 ka), almost all studied faults, excluding the Mugırtepe fault, experienced seismic events that resulted in prominent displacement. *Activity Phase IV* (ca. 2 ka) is associated with the youngest modeled seismic events that are remarkably time-correlated, similar to those of Activity Phase II. Therefore, the Manastır, Yavansu, Priene-Sazlı, and Ören faults seem to have ruptured simultaneously. Among these, the activity of Manastır (as a part of Manisa Fault Zone) and Priene-Sazlı faults correlate well with the historical earthquake records at 17 AD and 68 AD, respectively. By defining these activity phases, we do not assert that the region was completely dormant during the recurrence intervals, but that it was less active with only smaller earthquakes. Furthermore, it is not claimed that the faults activation occurred at a specific time with the exact correlation, but more showing a general comparison of seismic activity.

The investigated fault trends are mostly parallel/sub-parallel, with the exception of the Rahmiye fault, which tends to merge to the NNW-SSE Akselendi fault along most of its length (Figs. 1 and 2). This fault’s seismic activity seems to be synchronized with that of the other faults of interest only during Activity Phase III. We assume this distinction is due to the Rahmiye fault acting as the eastern boundary of the Gediz-Alaşehir Graben, its eastern trend contrasting with the general E-W trends of the graben systems.

### Length of recurrence intervals

Western Anatolia accommodates 122 fault segments, of which over 80% are characterized as normal dip-slip faults (Duman et al., 2018). Unfortunately, knowledge about most of these faults in terms of their activity histories, displacement characterization and the way they are linked to the whole tectonic network are very poor or even unknown. However, to enable a regional scale comparison of the seismic behavior of the faults that we did explore during the last decade (Akçar et al., 2012; Mozafari, et al., 2019; Mozafari, et al., 2019, 2021) the slip rates and recurrence intervals of major earthquakes for a series of normal faults located within the extensional regime of the Aegean Sea, as well as Israel and Italy are given in Table 6.

**Table 6:** Recurrence interval of major earthquakes of a number of faults within the extensional region of the Aegean sea and Italy

Location	Fault/Fault Zone	Fault/Fault Zone Length	Slip Rate (mm/yr)	Recurrence Interval (yr)	References
Turkey	Büyük Menderes Fault Zone	12–30	0.96–1.76	250–1900	Altunel et al. (2009)
	Manisa fault zone	35		250–669	Özkaymak (2012)
	Erdogmus fault	12	0.5	910 ± 40	Gürboğa (2013)
	Bolvadin fault	16	0.64	995 ± 40	Özkaymak et al. (2019)
	Dinar fault	50	1.0	1500–2000	Altunel et al. (1999)
	Honaz fault	15	0.15–0.38		Özkaymak (2015)
	North Anatolian fault, southern branches (Havran-Balıkesir and Edremit Fault Zones)	120	3.59–3.78	1000–2000	Sözbilir, et al. (2016), Sözbilir, et al. (2016))
	Sultanhanı fault, central Turkey	50	1.5 & 8.5	800–900	Melnick et al. (2017)
Greece	Spili fault (Crete)	20	0.6	4200	Mouslopoulou et al. (2014)
	Southern Lanada-Volvi Basin Margin Fault Zone	20		7000	Cheng et al. (1994)
	Sparta fault	150	0.5–2.0	500–4500	Benedetti et al. (2002)
	Atalanti fault	30	0.4–1.6	660–1120	Pantosti et al. (2004)
	Southern Mygdonia marginal fault (Gerakarou segment)	12	0.03–0.06	7000	Pavlides (1996)
	Tyrnavos fault (northern Thessaly)	25	0.1–0.2	2000–2500	Caputo et al. (2004)
	Pisia fault	25	0.5–2.3	1000 ± 500	Mechernich et al. (2018)
	Skinos fault	8	0.7–2.5	330	Collier et al. (1998)
	Kaparelli fault (Gulf of Corinth)	> 12	0.3	2500	Kokkalas et al. (2007)
	Kaparelli fault Mygdonia fault Eliki fault	12 50 25	0.3 0.26–0.7 1.5	2300 900 400–900	Chatzipetros et al. (2005)
	Gyrtoni fault	13	0.41	1400	Tsodoulos et al. (2016)
	Kaparelli fault	10	0.2	3000–9000 4000–5000 10,000–11,000	Benedetti et al. (2003)
	A series of normal faults (Crete)	9–16	0.5–2.3	260–840	Caputo et al. (2006)
Italy	Ovindoli–Pezza fault	> 20	0.9–2.5	1000–3000	Pantosti et al. (1996)
	Magnola fault	45	0.8	1000–3000	Palumbo et al. (2004)
	Fucino fault system	12–45	0.2–1.3	2000–3000	Benedetti et al. (2013)
	Velino-Magnola fault	45	1–1.5	1000–6000	Schlagenhauf et al. (2011)
	Campo Felice fault Roccapreturo fault	15 21	0.84–1.61 0.35–0.55	631 ± 620 2603 ± 1355	Goodall et al. (2021)
Israel	Nahef East fault	6	0.03	3000	Mitchell et al. (2001)

A wide range of recurrence intervals for earthquakes (hundreds to millions of years) associated with numerous faults has been reported worldwide (e.g., Nicol et al., 2005; Scholz, 2010; Sieh, 1981; Wallace, 1981). In several tectonic settings a regular pattern of earthquake recurrence intervals and identical slip rate values validates the concept of regional recurrence intervals (Scholz, 2010; Sieh, 1981; Wallace, 1981). In concordance with this concept, many researchers suggest that the recurrence interval of earthquakes on faults is primarily linked to the regional strain rate and fault size, and only secondarily to fault interactions (e.g., Nicol et al., 1997, 2005; Walsh et al., 2001). Nicol et al. (2005) formerly asserted that, in general, recurrence intervals of earthquakes in a given fault system are approximately identical for faults of roughly similar size. Certainly, only a general comparison with the faults in the whole region is possible because of the small number of existing data in the region with respect to the number of faults. Keeping this in mind, we tentatively conclude that both the Aegean extensional region and Italy, which are strongly controlled by normal faults with lengths of several tens of kilometers, are governed by recurrence intervals of major earthquakes on many faults of 1–5 kyr in average (Tables 5 and 6).

### Slip rates evaluation

**Fig. 15.**
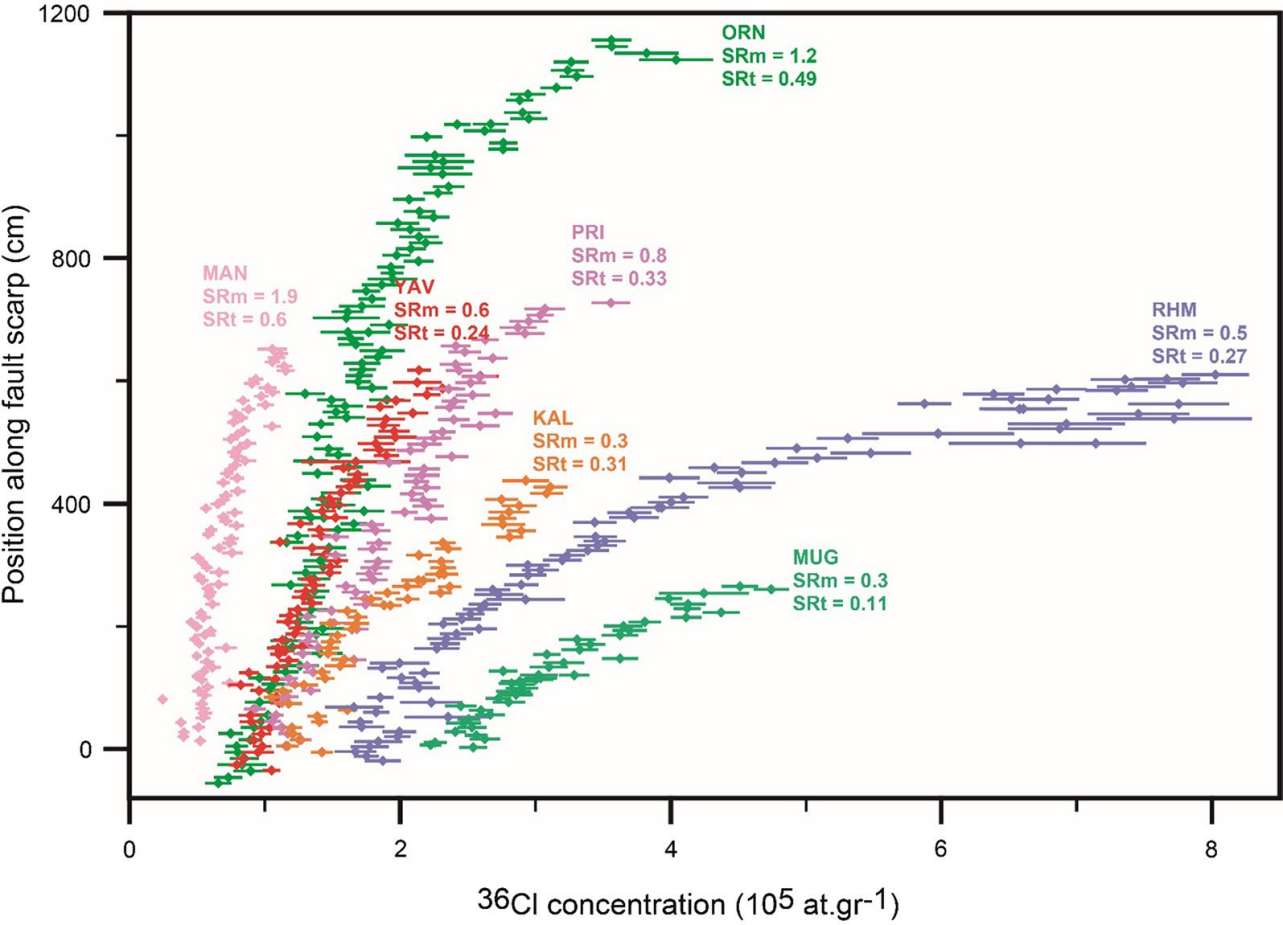
^36^Cl concentration with uncertainties measured for fault scarp dating of Rahmiye (RHM), Mugırtepe (MUG), Manastır (MAN), Kalafat (KAL), Yavansu (YAV), Priene-Sazlı (PRI) and Ören (ORN) faults. SRm defines average slip rates based on fault scarp modeling, while SRt represents theoretical average slip rates calculated based on scarp height dividing by mean LGM timing. The total scarp height of MAN, KAL, YAV and PRI faults are 12, 6.2, 7.2 and 8.5, respectively; however, the upper part of the faults remained unsampled due to being highly weathered and/or eroded

Hypothetically, cosmogenic ^36^Cl concentrations is subject to average slip rate of fault, which characteristically increase from base to top of the scarp surface (e.g., Schlagenhauf et al., 2010). Faster the fault slips, less curvature pattern of ^36^Cl concentrations forms (cf. Cowie et al., 2017; Schlagenhauf et al., 2010). Though, the time needed for an ideal exponential pattern of ^36^Cl concentrations below colluvium to be made before exposure is deficient. Accordingly, the build-up ^36^Cl concentrations plot of more active fault defines a higher slope compared to that of with less activity. Consequently, by plotting ^36^Cl concentrations values of faults of diverse slip rates a ‘fanning pattern’ is obtained (cf. Cowie et al., 2017). In order to evaluate consistency of our results with this theory, we collectively plotted 584 measurements of cosmogenic ^36^Cl concentration with corresponding uncertainties from the seven studied faults of western Anatolia (Fig. 15). The slip rates derived from the fault scarp modeling (SRm) are adequately consistent with this hypothesis (Fig. 15). However, for an additional source of approval, we approximated theoretical slip rate (SRt) by dividing total scarp height by the average 20 ka age of the Last Glacial Maximum (LGM) in Turkey (e.g., Akçar et al., 2017 among others), when demise of glaciers caused the minimizing of erosion and sedimentation rates, though well preservation of fault scarps. Theoretical slip rates at a high extent agree with the ‘fanning pattern’ representing values being decreased from the faults showing higher slope of cosmogenic ^36^Cl concentration to that of lower slope (Fig. 15). We attribute the negligible irregularity to varying dip of the faults and incomplete cosmogenic ^36^Cl analysis of four of the seven faults due to high erosion and/or weathering in the upper few meters of the scarp (Mozafari, et al., 2019; Mozafari, et al., 2019, 2021). The above statements, in addition to the field geomorphological evidence, provide solid indicators regarding validity of our method and demonstrate that these faults have been exposed to the surface in response to tectonic activities, not any other exhumation phenomena.

The inferred slip rates from fault scarp dating studies, at a high degree is consistent with the range of slip rates of normal faults in a regional scale (Tables 5 and 6). However, for a comprehensive understanding of fault behavior of a system, detailed slip rate assessment of faults in different time frames, because of its spatial and temporal fluctuation nature is efficient. Although not focus of this study, we made a brief comparison of short-term slip rate, using geodetic results of 17 years (Reilinger et al., 2006) and 8 years (Aktug et al., 2009) long (Table 7) with long-term slip rate utilised by displaced geological features after demise of LGM (Tables 5 and 6). Aktug et al. (2009), applying block division modeling and geodetical data, declared that the total extensional motion of 20 mm/yr of western Anatolia is partitioned along the large-scale faults. In our focused area, the dip-slip rate values determined to be varying from 14.5 to 20.2 mm/yr (Reilinger et al., 2006), at least triple the quantified value of 1.3 to 6.6 mm/yr (Aktug et al., 2009), owing increased number of blocks, which are set for the velocity measurements. In either approach, the greatest slip rate values have been distributed along the border of three grabens of interest, Gökova, Gediz-Alaşehir and Büyük Menderes in descending order (Table 7). Similar to many circumstances worldwide, slip rates in western Anatolia dramatically vary over geological and geodetical time windows (cf. Goodall et al., 2021; Evans et al., 2016; Faure Walker et al., 2010; Cowie et al., 2017; Dolan et al., 2007, 2016). Only, the lower limit of slip rates derived from geodetic data (Aktug et al., 2009) are to some degree consistent with incremental slip rates yielded from offsetting geological features (Tables 5 and 6). This is, at a high extent because the yielded geodetic slip rate is partitioned among the fault segments accommodated in the vicinity of GPS stations. Slip rate inconsistencies over geodetical and geological time spans could be in more detailed explained by several mechanism correspond to fluctuating rate of strain release such as varying characteristic of individual faults or fault zones, interaction of faults in a network scale, coulomb stress changes and post-seismic relaxation after earthquakes. However, discovering what is responsible for slip rate discrepancy in western Anatolia requires very detailed data beyond the aims of this work. The incremental slip rates also manifest that in western Anatolia, the faults reacted disparately over different earthquake cycles. In general, higher slip rates on individual faults through late Holocene, with some exceptions, implies either more activity of the faults or greater slip events (Table 5).

**Table 7:** Slip rates deduced from geodetical data

	Reilinger et al. (2006)	Aktug et al. (2009)
	GPS data (1988–2005)	GPS data (1997–2005)
	Avg. dip slip (mm/yr)	
Gediz-Alaşehir	16 ± 0.4	5.7
Büyük Menderes	14.5 ± 0.3	4.1
Gökova	20.2 ± 0.5	6.6

In the context of the regional tectonic regime, our results help constrain the Holocene deformation pattern in western Anatolia and its link to the convergence of the African plate. The African plate is being subducted beneath the Aegean plate, which is causing a shortening in the African plate parallel to the Hellenic arc resulting from thrust and strike-slip faulting, while extension is mostly evident on the Aegean plate through normal faulting, explicitly in western Anatolia and Greece (e.g., Angelier et al., 1982; Armijo et al., 1999; Jackson, 1994; McQuarrie et al., 2003; Shaw & Jackson, 2010). The rate of this convergence is still subject to debate, and values of 10 mm/yr (Oral et al., 1995) up to 40 mm/yr (Shaw & Jackson, 2010) have been reported for this northward motion. This large-scale movement results in a large amount of strain within the Aegean plate, which is partially accommodated on the faults, particularly on those with a parallel/sub-parallel orientation to the subduction trends. Shaw and Jackson (2010) state that a maximum of 20% of this motion is seismically accommodated, while most of the energy is aseismically consumed. The total strain, as mentioned above, will be partially loaded on the intraplate faults of the Anatolian plate, with the majority of it assumed to be transmitted along the largest faults (Cowie et al., 2007). The average slip rate of the studied faults only accounts for a small portion of the above mentioned rapid northward movement, however the partial strain accumulation on every discrete fault cannot be determined using the fault scarp dating method. The record of the recurrent large earthquakes together with high geodetical velocity may indicate that western Anatolia in the current time has been experiencing a release of the highest amount of accumulated strain during the Holocene. Accordingly, our findings might imply that we are now in a period of clustering earthquakes. Nevertheless, fault seismic activity is not merely connected to the localization of strain at a regional scale, but is also affected by a variety of factors, namely the local pattern of the nearby fault network, heterogeneity of the bedrock, degree of strength of the fault zone, and viscosity or viscoelastic condition of the lower crust (Cowie et al., 2007). While these factors add a further layer of complication to understanding fault behavior, our study provides valuable insight into the seismic activity of the complex western Anatolian tectonic regime.

## Conclusions

We have modeled paleoseismic histories of two normal fault scarps using ^36^Cl concentrations and compared these new measurements with previously published ^36^Cl data on other five nearby faults (Akçar et al., 2012; Mozafari, et al., 2019; Mozafari, et al., 2019, 2021) to provide a regional picture of the paleoseismicity of western Anatolia. At least 20 seismic events were inferred from seven faults (Fig. 1), mostly as clustered earthquakes close in time, with maximum magnitude of 6.5–7.1. Four synchronous phases of activity at around 2, 4, 6, and 8 ka indicate a recurrence interval of ca. 2000 years for high seismic activity on the regional scale of western Anatolia. This recurrence interval can be, generally compared with those inferred for other parts of the extensional region of the Aegean and Italy. Along with these seismic periods, several major normal faults in western Anatolia have been activated simultaneously, namely the 2017/02/07 MW = 5.3 Ayvacık, 2017/04/21 MW = 5.0 Manisa, 2017/05/28 MW = 4.9 Gölmarmara, 2017/06/12 MW = 6.4 Midilli, 2017/07/20 (M_W_ = 6.6) Bodrum, and 2017/11/24 MW = 5.3 Muğla earthquakes. The mean incremental slip rates of the western Anatolia faults analyzed in this study were calculated to be in the range of greater than 0.1 to 2.2 mm/yr and generally increase through time. These values are in the range of that of derived from nearly all the normal faults in the regional scale. Comparison of slip rates of our study shows that the faults acted differently through time in terms of either producing a greater number of earthquakes or larger displacement ensuing a single event, mostly during late Holocene. The average slip rates, however, vary in a range of 0.3 to 1.9 mm/yr. Geologic-geodetic slip rate comparison indicates obvious discrepancies correspond to varying strain accumulation, like many other global instances. The outcomes of recent studies on fault scarp dating provide informative research that enables a better understanding of the complex seismic behavior of western Turkey, which is essential to minimize probable damages of future earthquakes in this seismic-prone region.

## Supplementary Information

**Additional file 1: Figure S1**. Schematic sketch of cosmogenic ^36^Cl profile. **Figures S2–S10.** Additional field photos.

**Additional file 2: Table S1. **Regression of magnitude (MS/M), maximum vertical displacement (MVD/MD), SRL (surface rupture length).** Table S2.** Stable Cl, cosmogenic ^36^Cl, calcium, oxygen and carbon concentrations, thickness, top and bottom position of the samples from the Rahmiye scarp. **Table S3.** Stable Cl, cosmogenic ^36^Cl, calcium, oxygen and carbon concentrations, thickness, top and bottom position of the samples from the Ören scarp. **Table S4.** Blank measurements along with associated samples processed in similar batches. **Table S5.** Mean chemical composition of the Rahmiye fault scarp samples and colluvium. **Table S6.** Mean chemical composition of the Ören fault scarp samples and colluvium. **Table S7.** Output results of the lowest statistical criterion for different rupture histories of the faults.

**Additional file 3. **Fault Scarp Dating Tool (FSDT), the Matlab® code developed by Tikhomirov et al. (2019) along with Rahmiye and Ören faults datasets to run the program have been provided.

## Data Availability

Not applicable.
