# Supplementary material for: Seismic history of western Anatolia during the last 16 kyr determined by cosmogenic 36Cl dating
Source: Swiss J Geosci. 2022 Feb 17;115(1):5. doi: 10.1186/s00015-022-00408-x (PMC8854328; doi:10.1186/s00015-022-00408-x)

**Appendix (Figures)**

Figure S1. Schematic sketch of cosmogenic ^36^Cl profile on both exposed and covered by colluvium fault surface following four earthquakes of similar size (After Mozafari et al., 2019a).


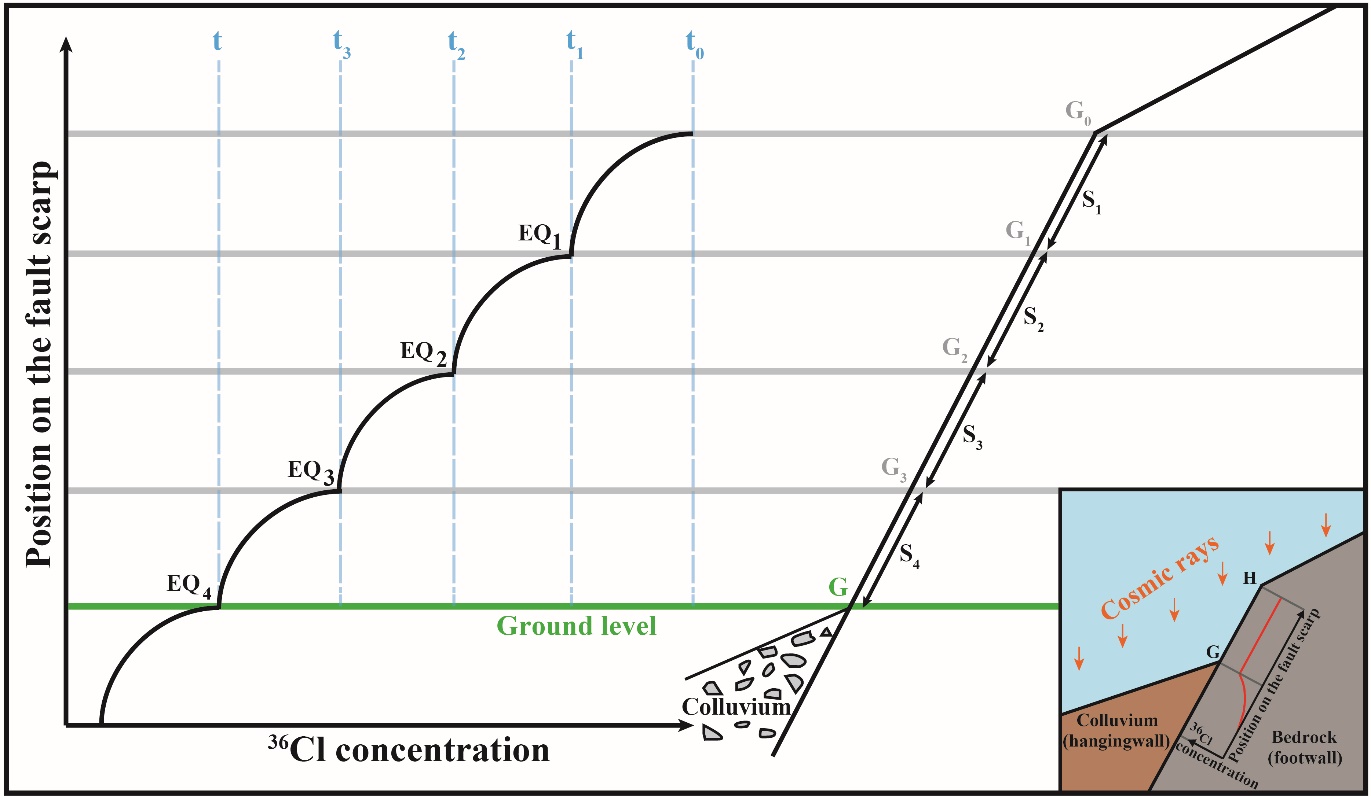

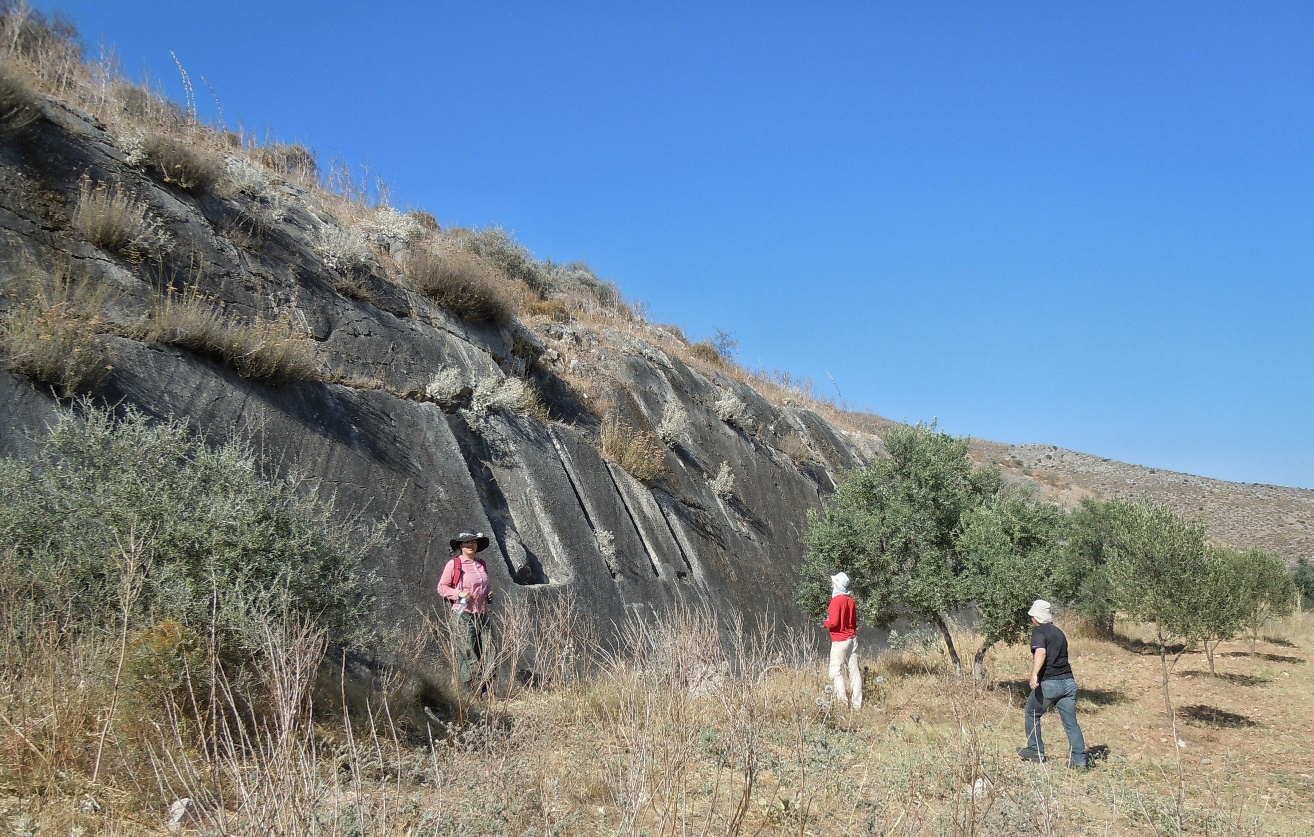


Figure S2. View of Rahmiye fault surface at the sampling site before sampling. The orange arrow denotes the profile to be sampled, also the scarp height (defines white dashed line in Fig. 3c).

Figure S3. using a ladder and/or scaffold to access the entire fault surface for sampling (a) Rahmiye fault; (b) Ören fault. The orange arrows denote the scarp height, also define white dashed line in Fig. 3c and 5b).


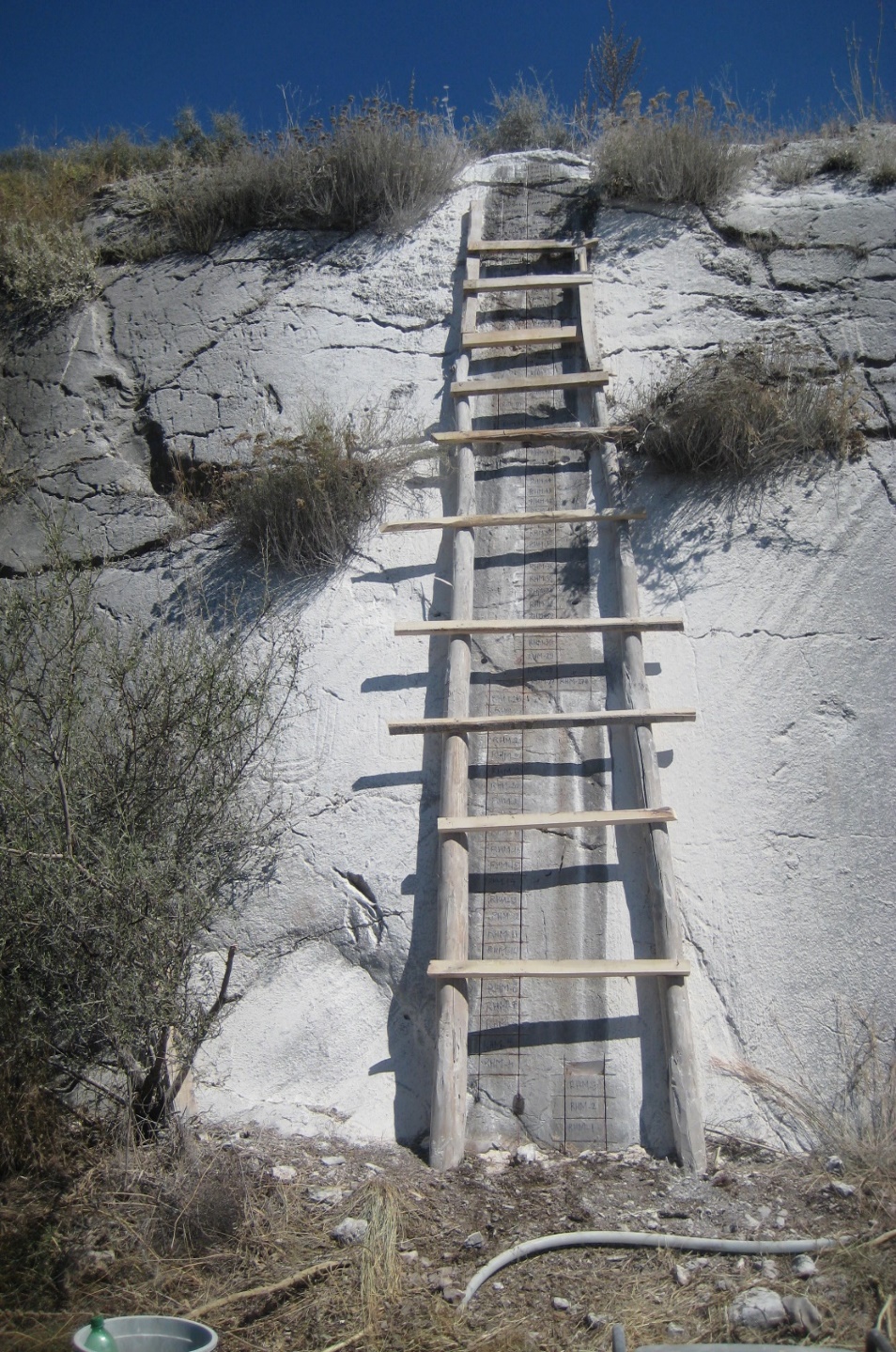

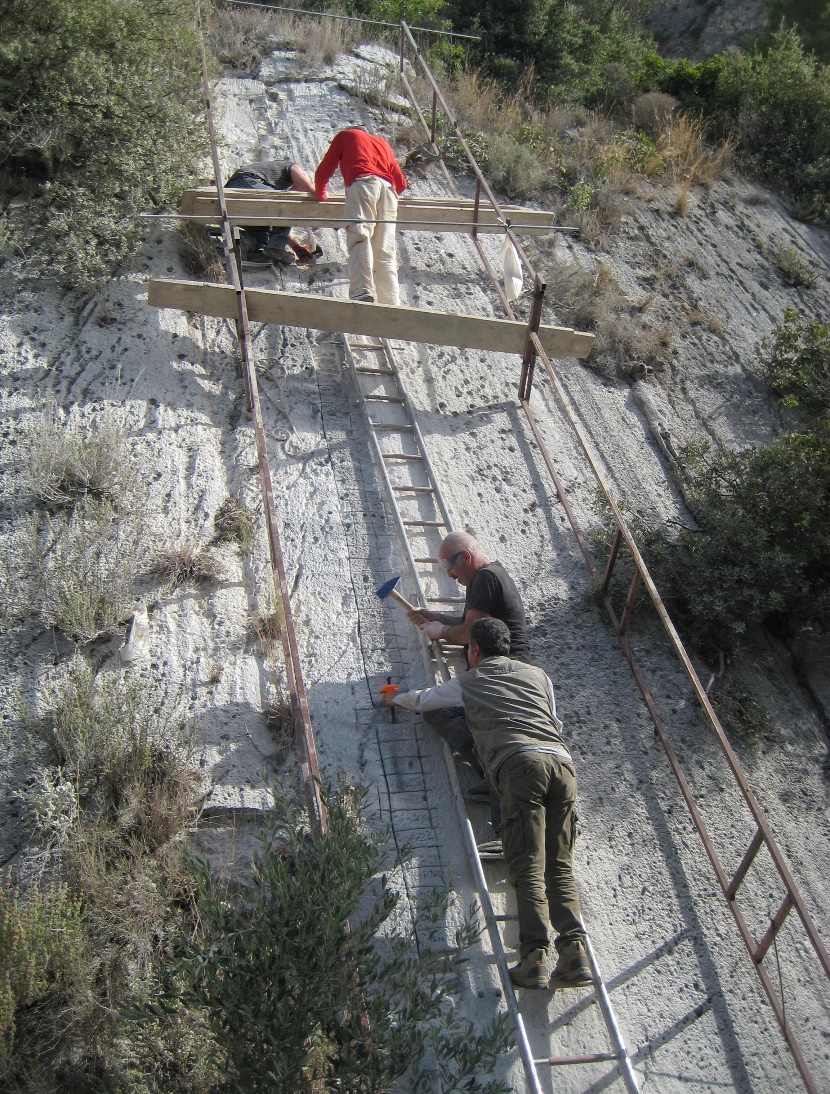


**(b)**

**(a)**


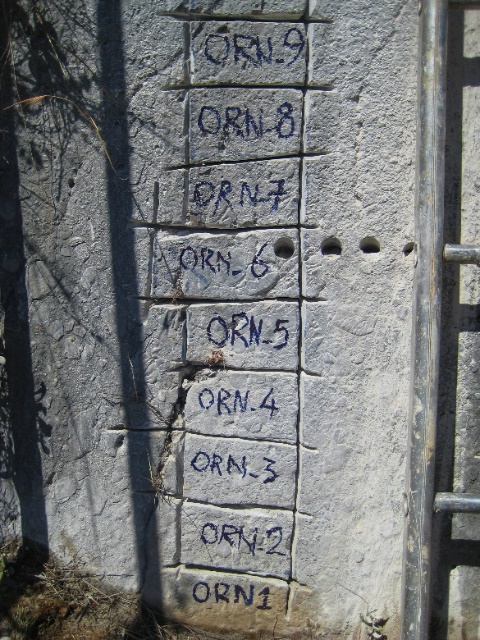

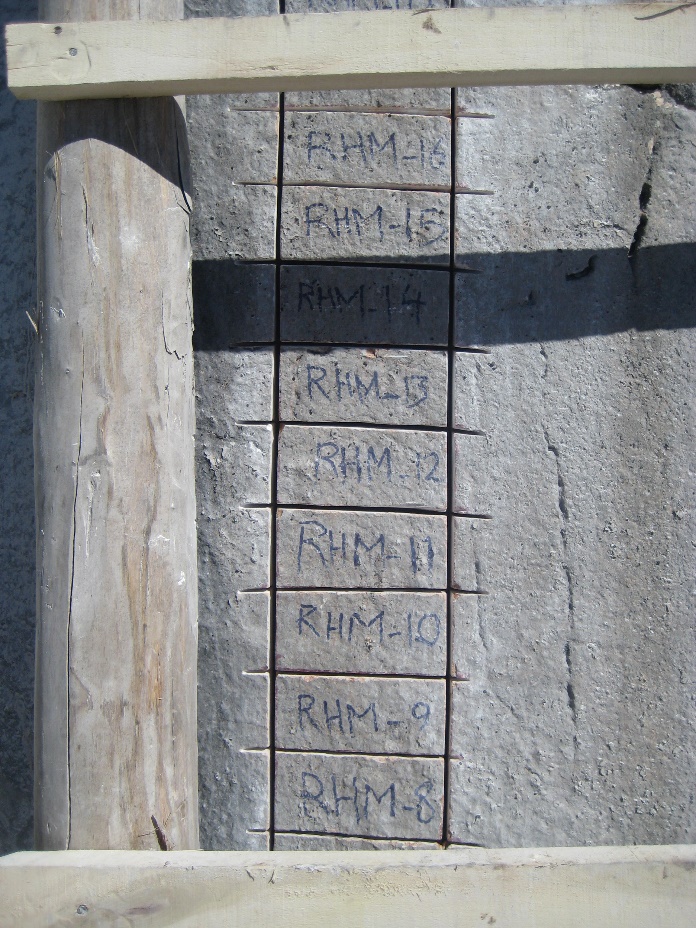


Figure S4. (a-b) Marked samples on the Rahmiye and Ören faults, respectively; (c-d) samples collection using chisel and hammer in Rahmiye and Ören faults, respectively.


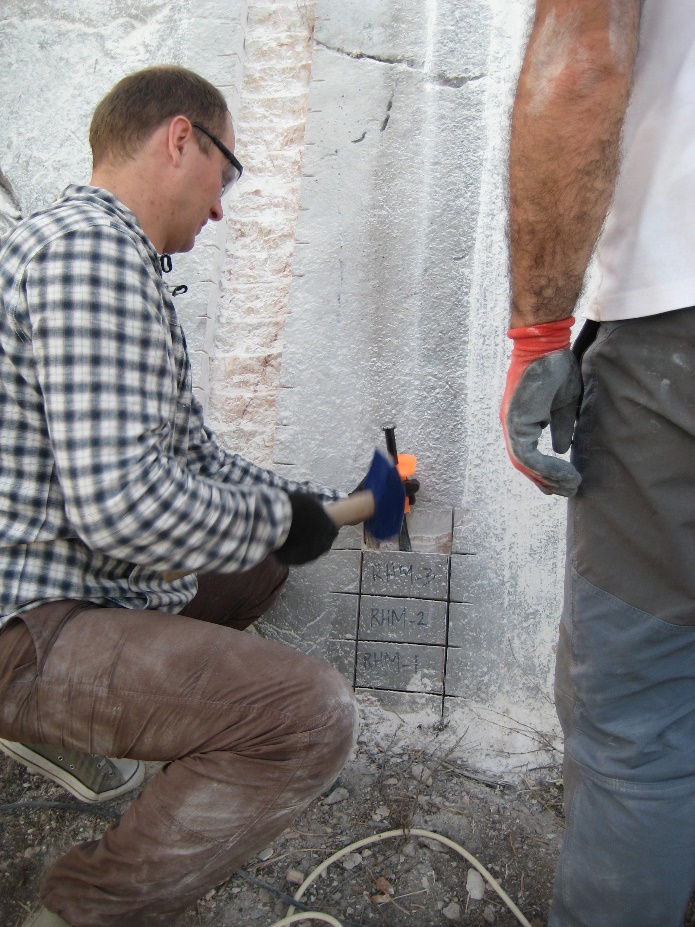

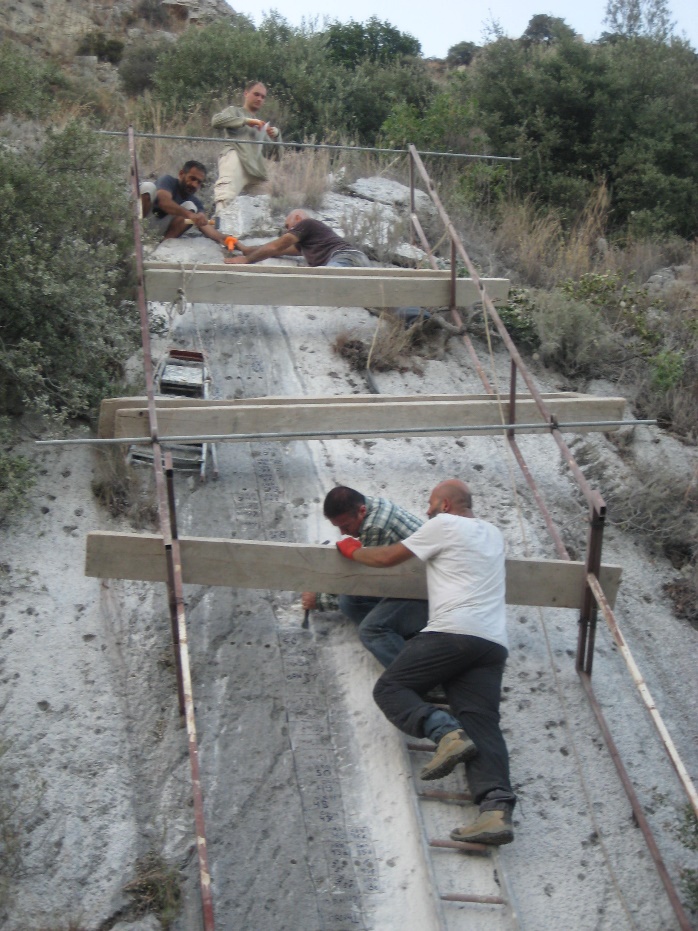


**(a)**

**(b)**

**(c)**

**(d)**

Figure S5. (a) Side view of Rahmiye fault surface at the sampling site. The gentle dip of colluvium is observable. The orange arrow denotes the profile to be sampled, also the scarp height. The cartoon shows the hypothetical line extrapolated from dip of colluvium to define ground level; (b) and (c) View of the excavated part of Rahmiye fault surface (sub-surface) from the east of sampling site (marked by star) towards WNW and ENE, respectively, showing colluvium gentle dip as the hangingwall.


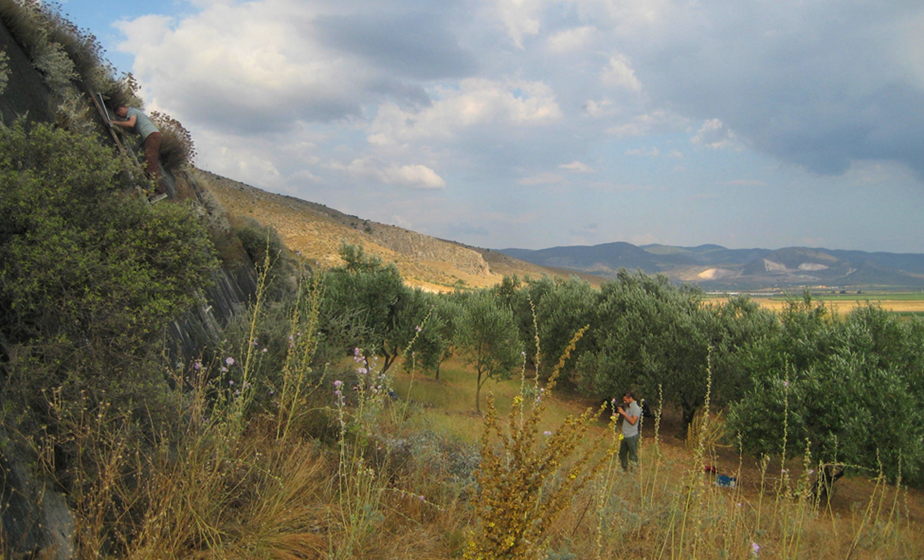

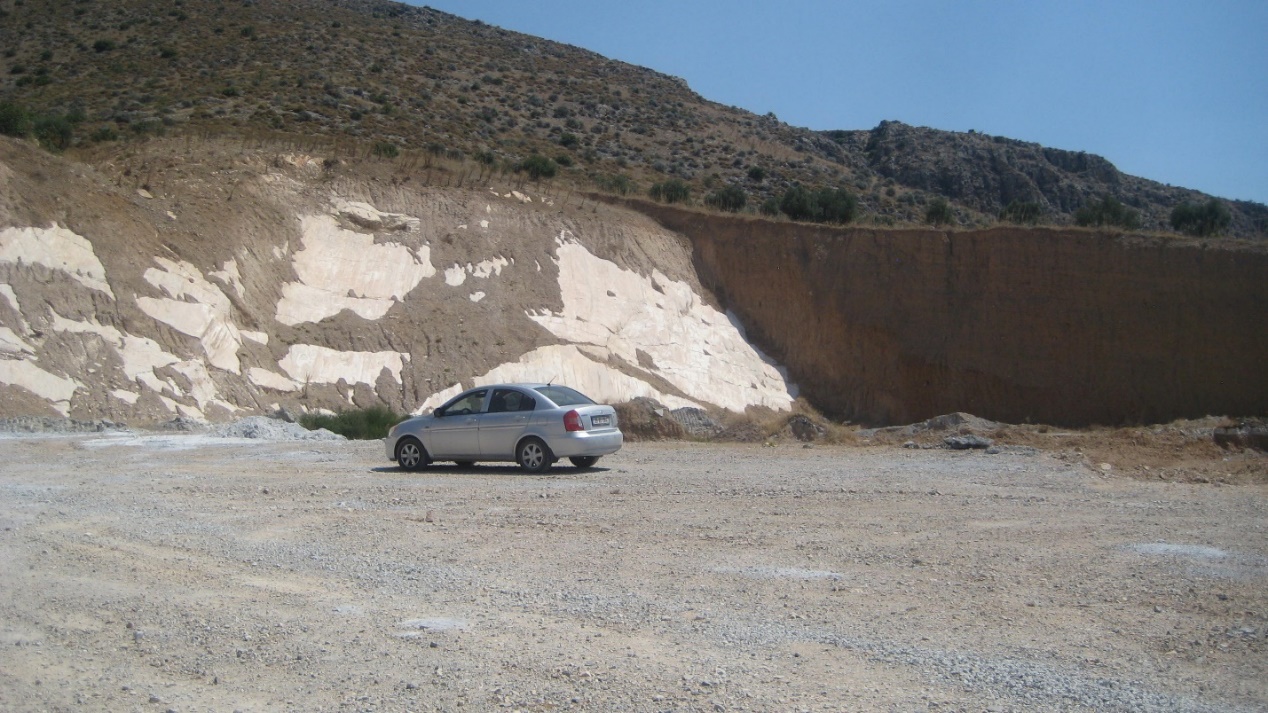

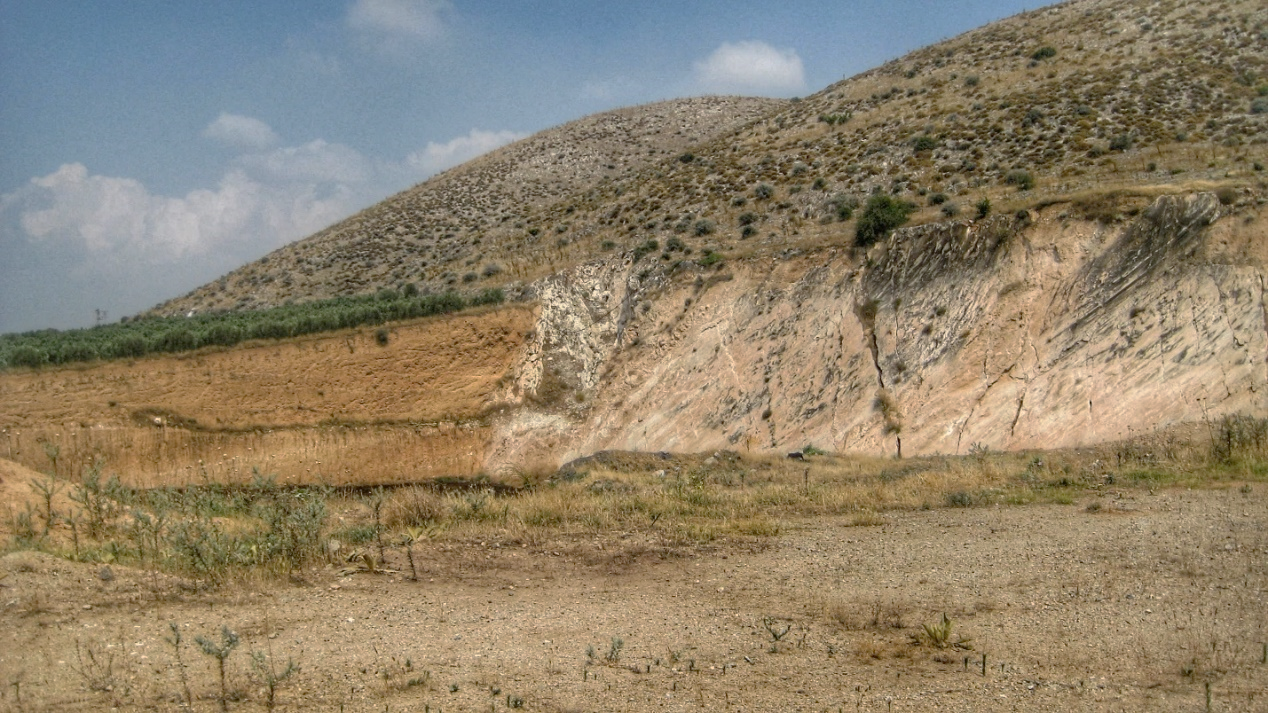


**(a)**

**(b)**

**N**

**(c)**

**WNW**

**ESE**

**ENE**

**WSW**

ground level

(for modeling)

Colluvium dip

current

ground level


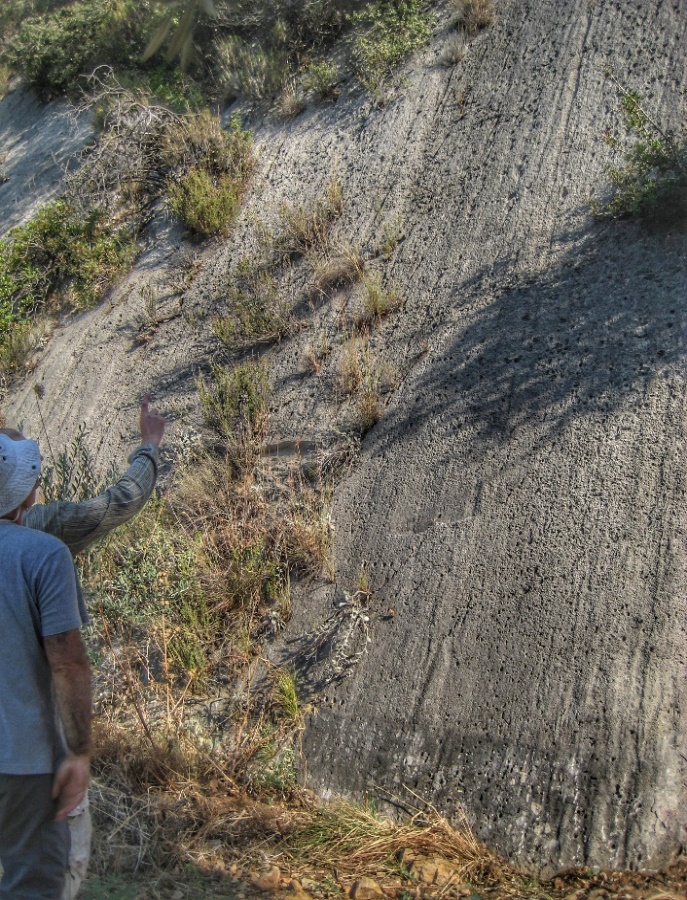


Figure S6. (a) Lower surface of the Ören fault. The arrow denotes the ground level, which borders previously covered (lower darker part) and the exposed sections of the fault. Dip-slip striations are clear on the fault surface.

Figure S7. View of Rahmiye fault showing triangle facets denoted by black arrows. The star marks the sampling site.


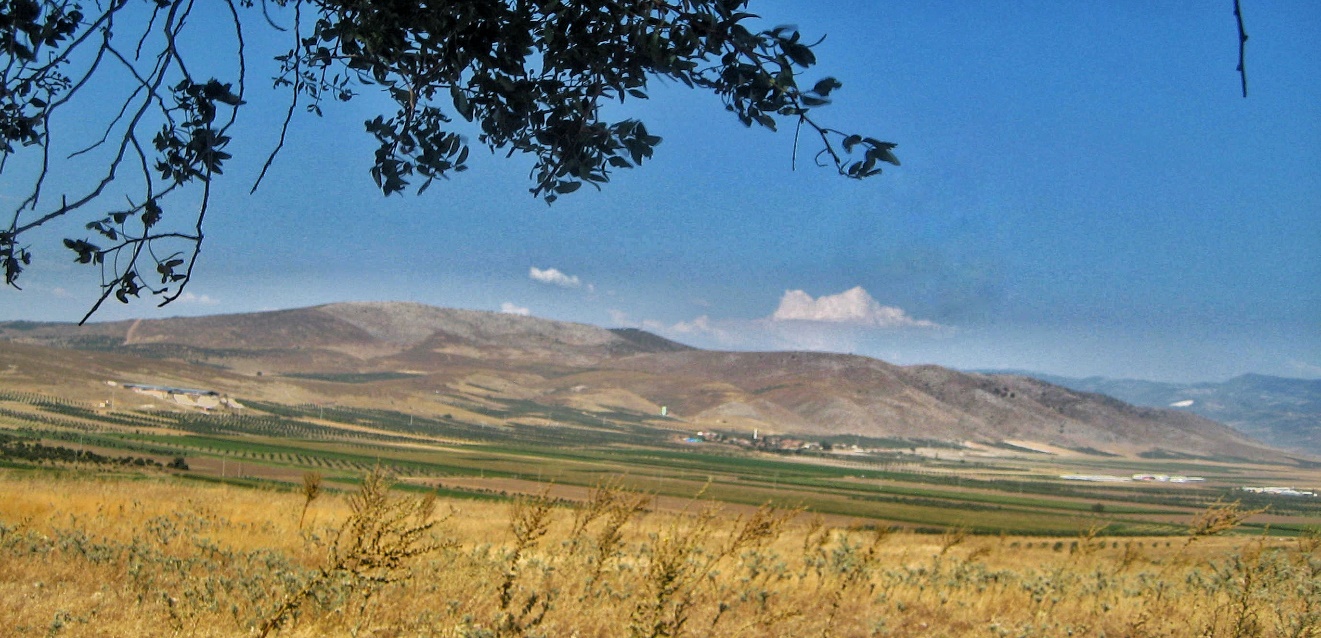


Figure S8. View of Rahmiye fault surface at the sampling site. Dissolution marks in the upper part of the fault surface, specifically in the upper right corner visible as straight lines. These parallel straight lines are totally missing in the lower part of the fault surface. Striations indicating dextral dip-slip movement are mainly observable in the lower part.


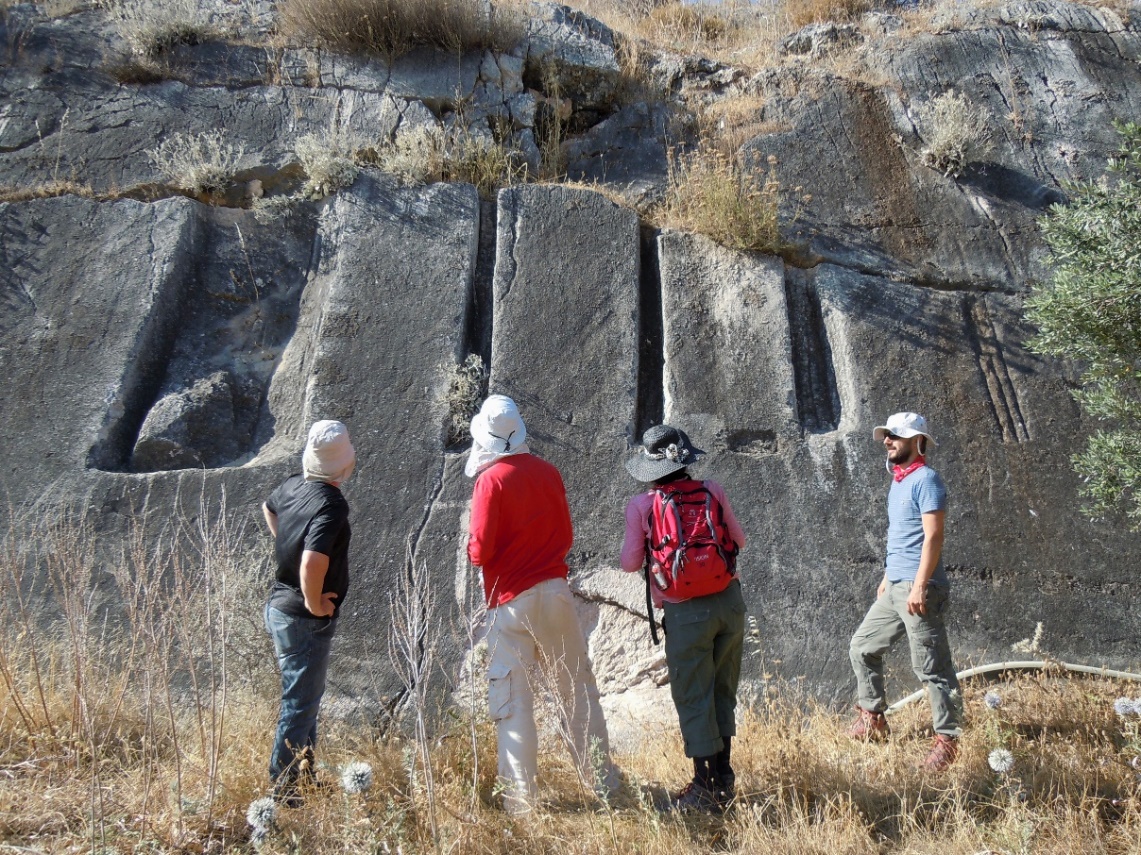


**Colluvium**

Figure S9. Solution space of three seismic event scenario for the Rahmiye fault populated with 102408 simulations and solution space of four seismic event scenario for the Ören fault populated with 107428 simulations including the best fit solutions of Rahmiye and Ören faults.


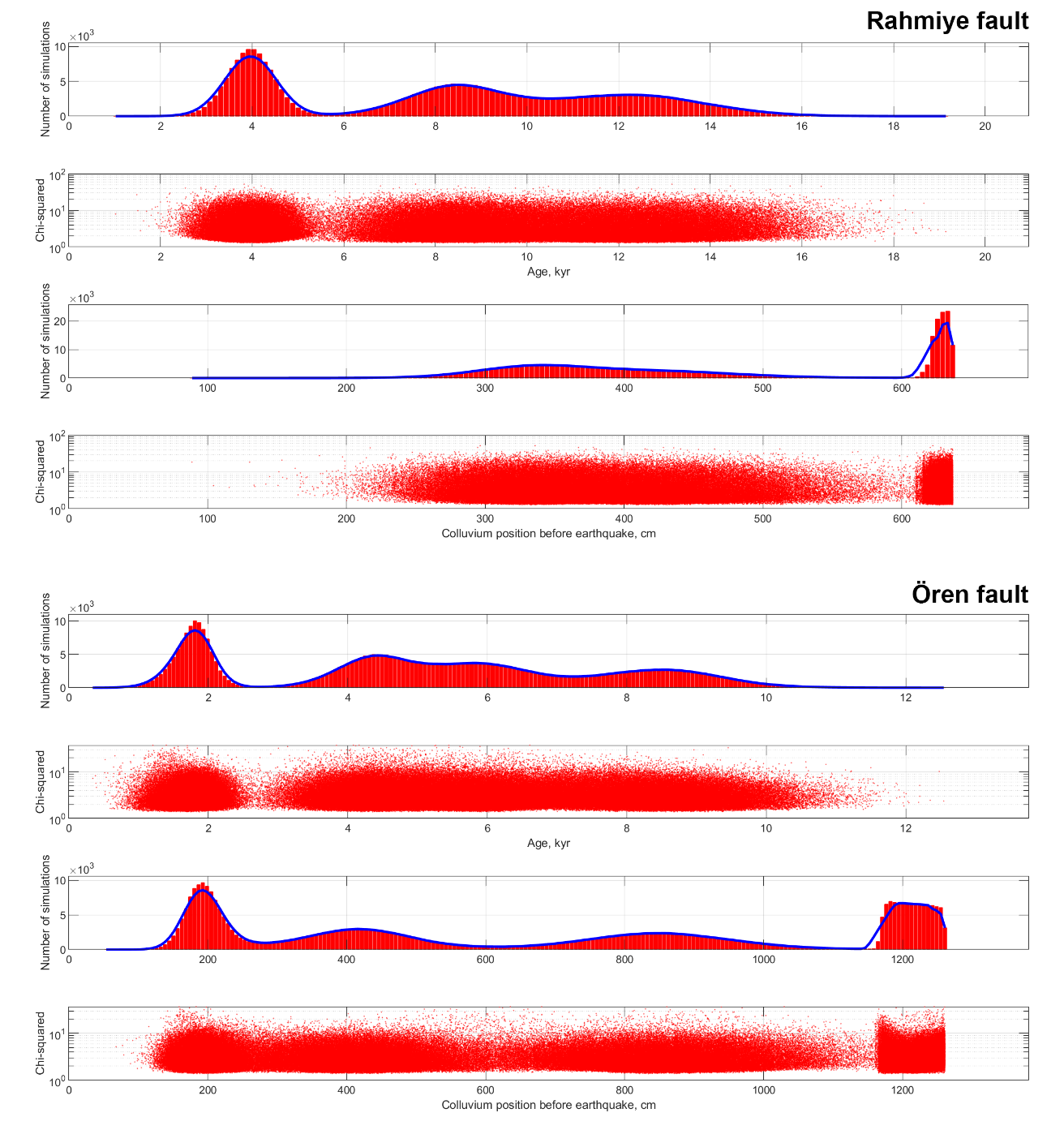

Supplement: Supplementary file 1 — Additional file 1: Figure S1. Schematic sketch of cosmogenic 36Cl profile. Figures S2–S10. Additional field photos. [file 15_2022_408_MOESM1_ESM.docx]
