# Supplementary material for: Seismic history of western Anatolia during the last 16 kyr determined by cosmogenic 36Cl dating
Source: Swiss J Geosci. 2022 Feb 17;115(1):5. doi: 10.1186/s00015-022-00408-x (PMC8854328; doi:10.1186/s00015-022-00408-x)
Supplement: Supplementary file 2 — Additional file 2: Table S1. Regression of magnitude (MS/M), maximum vertical displacement (MVD/MD), SRL (surface rupture length). Table S2. Stable Cl, cosmogenic 36Cl, calcium, oxygen and carbon concentrations, thickness, top and bottom position of the samples from the Rahmiye scarp. Table S3. Stable Cl, cosmogenic 36Cl, calcium, oxygen and carbon concentrations, thickness, top and bottom position of the samples from the Ören scarp. Table S4. Blank measurements along with associated samples processed in similar batches. Table S5. Mean chemical composition of the Rahmiye fault scarp samples and colluvium. Table S6. Mean chemical composition of the Ören fault scarp samples and colluvium. Table S7. Output results of the lowest statistical criterion for different rupture histories of the faults. [file 15_2022_408_MOESM2_ESM.docx]

**Appendix (Tables)**

Table A1. Regression of magnitude (MS/M), maximum vertical displacement (MVD/MD), SRL (surface rupture length) used for calculations

| Pavlides & Caputo (2004) | equation 1 | Ms = 0.9 x Log (SRL) + 5.48 |
| --- | --- | --- |
|  | equation 2 | Log (MVD) = 1.14.Ms - 7.82 |
|  | equation 5 | Ms = 0.59 x Log (MVD) + 6.75 |
| Wells & Coppersmith (1994) | equation 3 | M = 4.86 + 1.32 x log (SRL) |
|  | equation 4 | Log (MD) = - 5.90 + 0.89 x M |
|  | equation 6 | M = 6.61 + 0.71 x log (MD) |

Table A2. Stable Cl, cosmogenic ^36^Cl, calcium, oxygen and carbon concentrations, thickness, top and bottom position of the samples from the Rahmiye fault scarp

| Sample name | Top position  (cm) | Bottom position  (cm) | Sample  thickness  (cm) | ^36^Cl*  (10^5^ at/g) | ^36^Cl uncertainty*  (10^5^ at/g) | Cl total*  (ppm) | Cl total uncertainty*  (ppm) | Ca✝  (%) | O  (%) | C  (%) |
| --- | --- | --- | --- | --- | --- | --- | --- | --- | --- | --- |
| RHM-1 | -15.2 | -23.6 | 3 | 1.872 | 0.123 | 1.76 | 0.02 | 39.10 | 48.84 | 11.59 |
| RHM-2 | -7.8 | -15.6 | 3 | 1.752 | 0.082 | 1.33 | 0.01 | 38.90 | 49.18 | 11.53 |
| RHM-3 | 0 | -8.3 | 3 | 1.671 | 0.145 | 1.11 | 0.01 | 38.90 | 49.14 | 11.53 |
| RHM-4 | 7.8 | 0 | 3 | 1.776 | 0.130 | 1.84 | 0.02 | 38.80 | 49.23 | 11.50 |
| RHM-5 | 16 | 8.2 | 3 | 1.838 | 0.167 | 1.17 | 0.01 | 39.50 | 48.43 | 11.71 |
| RHM-6 | 24 | 16.4 | 3 | 1.981 | 0.087 | 1.01 | 0.01 | 38.90 | 49.15 | 11.53 |
| RHM-7 | 31.8 | 24.4 | 3 | 1.995 | 0.113 | 1.50 | 0.01 | 39.60 | 48.22 | 11.74 |
| RHM-8 | 40 | 32.2 | 3 | 1.716 | 0.157 | 1.07 | 0.01 | 38.60 | 49.50 | 11.44 |
| RHM-9 | 47.7 | 40.3 | 3 | 1.706 | 0.082 | 1.11 | 0.01 | 38.70 | 49.33 | 11.47 |
| RHM-10 | 55.9 | 48 | 3 | 2.355 | 0.313 | 1.37 | 0.01 | 39.00 | 48.98 | 11.56 |
| RHM-11 | 63.8 | 56.3 | 3 | 1.823 | 0.085 | 1.55 | 0.02 | 39.00 | 48.98 | 11.56 |
| RHM-12 | 72 | 64.2 | 3 | 1.659 | 0.204 | 1.51 | 0.02 | 39.30 | 48.60 | 11.65 |
| RHM-13 | 79.8 | 72.5 | 3 | 2.231 | 0.220 | 3.00 | 0.03 | 39.30 | 48.50 | 11.65 |
| RHM-14 | 87.8 | 80.2 | 3 | 1.853 | 0.087 | 1.24 | 0.01 | 39.20 | 48.78 | 11.62 |
| RHM-16 | 103.5 | 96.2 | 3 | 2.139 | 0.144 | 2.52 | 0.03 | 39.00 | 48.95 | 11.56 |
| RHM-17 | 111.6 | 104 | 3 | 2.116 | 0.111 | 1.67 | 0.02 | 39.40 | 48.48 | 11.68 |
| RHM-18 | 120 | 112.3 | 3 | 2.010 | 0.097 | 2.72 | 0.03 | 39.00 | 48.77 | 11.56 |
| RHM-19 | 127.7 | 120.4 | 2.5 | 2.178 | 0.102 | 1.89 | 0.02 | 38.70 | 49.33 | 11.47 |
| RHM-20 | 135.6 | 128.1 | 3 | 1.869 | 0.099 | 1.13 | 0.01 | 38.90 | 49.14 | 11.53 |
| RHM-21 | 143.6 | 136 | 3 | 1.996 | 0.210 | 1.29 | 0.01 | 38.80 | 49.25 | 11.50 |
| RHM-24 | 167.4 | 160.3 | 3 | 2.271 | 0.157 | 0.91 | 0.01 | 39.40 | 48.58 | 11.68 |
| RHM-25 | 175.7 | 168 | 3 | 2.332 | 0.097 | 0.62 | 0.01 | 39.60 | 48.31 | 11.74 |
| RHM-26 | 183.6 | 176.2 | 2.5 | 2.343 | 0.133 | 1.10 | 0.01 | 38.70 | 49.42 | 11.47 |
| RHM-27 | 191.5 | 184 | 3 | 2.417 | 0.111 | 1.77 | 0.02 | 38.20 | 49.93 | 11.33 |
| RHM-28 | 199.6 | 192 | 3 | 2.584 | 0.121 | 1.33 | 0.01 | 38.90 | 49.13 | 11.53 |
| RHM-29 | 207.7 | 200.1 | 3 | 2.319 | 0.101 | 1.30 | 0.01 | 39.20 | 48.76 | 11.62 |
| RHM-30 | 215.5 | 208.1 | 3 | 2.456 | 0.131 | 3.91 | 0.04 | 39.40 | 48.39 | 11.68 |
| RHM-31 | 224.1 | 216 | 3 | 2.520 | 0.097 | 2.07 | 0.02 | 39.30 | 48.62 | 11.65 |
| RHM-32 | 231.8 | 224.5 | 2.5 | 2.593 | 0.117 | 1.24 | 0.01 | 39.50 | 48.39 | 11.71 |
| RHM-33 | 239.8 | 232.3 | 3 | 2.626 | 0.110 | 2.16 | 0.02 | 39.70 | 48.05 | 11.77 |
| RHM-34 | 247.6 | 240.2 | 3 | 2.928 | 0.281 | 24.77 | 0.25 | 38.50 | 48.67 | 11.41 |
| RHM-35 | 255.5 | 248.2 | 2.5 | 2.729 | 0.170 | 1.31 | 0.01 | 39.80 | 47.95 | 11.80 |
| RHM-36 | 263.5 | 256 | 3 | 2.685 | 0.120 | 0.81 | 0.01 | 39.90 | 47.85 | 11.83 |
| RHM-37 | 271.8 | 264 | 3 | 2.895 | 0.116 | 0.72 | 0.01 | 39.50 | 48.28 | 11.71 |
| RHM-39 | 287.5 | 279.9 | 3 | 2.945 | 0.126 | 1.94 | 0.02 | 39.50 | 48.33 | 11.71 |
| RHM-40 | 295.6 | 288 | 3 | 3.038 | 0.125 | 1.81 | 0.02 | 39.20 | 48.76 | 11.62 |
| RHM-41 | 303.7 | 296.1 | 3 | 2.943 | 0.151 | 2.17 | 0.02 | 39.00 | 48.94 | 11.56 |
| RHM-42 | 311.8 | 304.1 | 3 | 3.199 | 0.133 | 1.52 | 0.02 | 39.10 | 48.90 | 11.59 |
| RHM-43 | 319.5 | 312.4 | 3 | 3.235 | 0.121 | 1.25 | 0.01 | 39.60 | 48.25 | 11.74 |
| RHM-44 | 327.6 | 320 | 3 | 3.386 | 0.146 | 1.83 | 0.02 | 39.90 | 47.89 | 11.83 |
| RHM-45 | 335.6 | 328.1 | 3 | 3.458 | 0.135 | 1.96 | 0.02 | 38.80 | 49.29 | 11.50 |
| RHM-46 | 342 | 336 | 3 | 3.510 | 0.144 | 1.88 | 0.02 | 39.90 | 47.89 | 11.83 |
| RHM-47 | 349.9 | 342.5 | 3 | 3.444 | 0.148 | 0.98 | 0.01 | 39.00 | 49.27 | 11.56 |
| RHM-48 | 373.5 | 365.7 | 3 | 3.438 | 0.150 | 0.75 | 0.01 | 39.70 | 48.15 | 11.77 |
| RHM-49 | 381.3 | 373.9 | 3 | 3.729 | 0.172 | 1.09 | 0.01 | 39.10 | 48.81 | 11.59 |
| RHM-50 | 389.3 | 381.7 | 3 | 3.692 | 0.152 | 1.32 | 0.01 | 38.70 | 49.34 | 11.47 |
| RHM-51 | 397.4 | 389.9 | 3 | 3.933 | 0.193 | 1.24 | 0.01 | 39.40 | 48.43 | 11.68 |
| RHM-51 | 397.6 | 390.1 | 3 | 3.909 | 0.197 | 1.73 | 0.02 | 39.40 | 48.37 | 11.68 |
| RHM-52 | 406.5 | 398.1 | 3 | 4.004 | 0.160 | 3.24 | 0.03 | 39.10 | 48.66 | 11.59 |
| RHM-53 | 414.7 | 406.9 | 3 | 4.095 | 0.171 | 2.95 | 0.03 | 39.10 | 48.66 | 11.59 |
| RHM-55 | 429.9 | 422.9 | 3 | 4.510 | 0.221 | 26.54 | 0.27 | 38.40 | 49.00 | 11.38 |
| RHM-56 | 437.3 | 430.4 | 3 | 4.485 | 0.277 | 75.03 | 0.75 | 36.00 | 50.31 | 10.67 |
| RHM-57 | 445.8 | 437.9 | 3 | 3.990 | 0.212 | 31.50 | 0.31 | 38.10 | 48.84 | 11.30 |
| RHM-58 | 453.8 | 446.3 | 3 | 4.525 | 0.173 | 8.49 | 0.08 | 38.80 | 48.95 | 11.50 |
| RHM-59 | 462 | 454.4 | 3 | 4.323 | 0.180 | 6.47 | 0.06 | 39.10 | 48.70 | 11.59 |
| RHM-59 | 469.1 | 463.1 | 3 | 4.769 | 0.234 | 23.72 | 0.24 | 38.50 | 48.85 | 11.41 |
| RHM-60 | 477.7 | 470.1 | 3 | 5.082 | 0.209 | 18.12 | 0.18 | 38.50 | 49.11 | 11.41 |
| RHM-61 | 485.6 | 478.2 | 3 | 5.478 | 0.287 | 22.86 | 0.23 | 37.40 | 49.79 | 11.09 |
| RHM-62 | 493.5 | 486 | 3 | 4.931 | 0.213 | 22.27 | 0.22 | 37.30 | 50.13 | 11.06 |
| RHM-63 | 502 | 493.9 | 3 | 7.142 | 0.364 | 104.01 | 1.04 | 35.20 | 50.80 | 10.44 |
| RHM-63 | 501.9 | 493.8 | 3 | 6.586 | 0.523 | 127.96 | 1.28 | 34.20 | 51.42 | 10.14 |
| RHM-64 | 509.7 | 502.3 | 2.5 | 5.308 | 0.217 | 40.35 | 0.40 | 36.40 | 50.86 | 10.79 |
| RHM-65 | 517.4 | 510.1 | 2 | 5.976 | 0.551 | 37.39 | 0.37 | 36.70 | 49.93 | 10.88 |
| RHM-66 | 525.5 | 517.8 | 3 | 6.875 | 0.371 | 108.72 | 1.09 | 35.00 | 50.78 | 10.38 |
| RHM-67 | 533.5 | 526 | 2.5 | 6.924 | 0.423 | 63.13 | 0.63 | 36.00 | 50.18 | 10.67 |
| RHM-68 | 541.8 | 533.9 | 3 | 7.720 | 0.562 | 114.66 | 1.15 | 34.60 | 50.57 | 10.26 |
| RHM-69 | 549.3 | 542.3 | 3 | 7.457 | 0.365 | 92.59 | 0.93 | 34.90 | 51.20 | 10.35 |
| RHM-70 | 558.5 | 549.7 | 3 | 6.605 | 0.314 | 56.87 | 0.57 | 36.00 | 50.78 | 10.67 |
| RHM-70 | 557.7 | 549.7 | 3 | 6.577 | 0.252 | 8.58 | 0.09 | 38.70 | 49.13 | 11.47 |
| RHM-71 | 565.8 | 559.3 | 2.5 | 5.875 | 0.187 | 4.03 | 0.04 | 40.00 | 47.56 | 11.86 |
| RHM-71 | 565.7 | 558.2 | 3 | 7.754 | 0.362 | 76.89 | 0.77 | 35.50 | 51.07 | 10.52 |
| RHM-72 | 574 | 566.3 | 2.5 | 6.518 | 0.202 | 6.18 | 0.06 | 39.30 | 48.38 | 11.65 |
| RHM-73 | 582 | 574.5 | 3 | 6.387 | 0.215 | 1.73 | 0.02 | 39.40 | 48.46 | 11.68 |
| RHM-74 | 586.1 | 582.5 | 3 | 7.295 | 0.221 | 4.91 | 0.05 | 39.30 | 48.44 | 11.65 |
| RHM-74 | 589.9 | 582 | 2.5 | 6.850 | 0.212 | 4.14 | 0.04 | 38.70 | 49.35 | 11.47 |
| RHM-75 | 573.3 | 565.9 | 3 | 6.791 | 0.217 | 7.37 | 0.07 | 36.80 | 51.70 | 10.91 |
| RHM-76 | 605.7 | 597.9 | 3 | 7.357 | 0.244 | 14.23 | 0.14 | 38.80 | 48.97 | 11.50 |
| RHM-77 | 593.1 | 587.2 | 3 | 7.405 | 0.242 | 1.46 | 0.01 | 39.50 | 48.38 | 11.71 |
| RHM-78 | 599.2 | 593.6 | 3 | 7.784 | 0.245 | 1.91 | 0.02 | 39.60 | 48.20 | 11.74 |
| RHM-79 | 606.1 | 599.6 | 3 | 7.666 | 0.235 | 2.51 | 0.03 | 39.60 | 48.19 | 11.74 |
| RHM-80 | 613.1 | 606.7 | 3 | 8.025 | 0.238 | 1.73 | 0.02 | 39.10 | 48.85 | 11.59 |
| * Measured with accelerator mass spectrometry (AMS).  ✝Measured with ICP in Actlabs Analytical services, Canada. | | | | | | | |  |  |  |

Table A3. Stable Cl, cosmogenic ^36^Cl, calcium, oxygen and carbon concentrations, thickness, top and bottom position of the samples from the Ören fault scarp

| ample name | Top position  (cm) | Bottom position  (cm) | Sample thickness  (cm) | ^36^Cl*  (10^5^ at/g) | ^36^Cl uncertainty*  (10^5^ at/g) | Cl total*  (ppm) | Cl total uncertainty*  (ppm) | Ca✝  (%) | O  (%) | C  (%) |
| --- | --- | --- | --- | --- | --- | --- | --- | --- | --- | --- |
| ORN-1 | -51.8 | -59.8 | 3 | 0.656 | 0.086 | 3.93 | 0.04 | 38.9 | 48.87 | 11.53 |
| ORN-2 | -41.5 | -51.5 | 3 | 0.730 | 0.094 | 3.98 | 0.04 | 38.9 | 48.76 | 11.53 |
| ORN-3 | -30.7 | -41 | 3 | 0.895 | 0.116 | 3.73 | 0.04 | 39 | 48.68 | 11.56 |
| ORN-4 | -20.7 | -30.3 | 3 | 0.831 | 0.171 | 4.51 | 0.05 | 38.5 | 49.47 | 11.41 |
| ORN-5 | -10.7 | -20.1 | 3 | 0.849 | 0.095 | 4.36 | 0.04 | 39 | 48.66 | 11.56 |
| ORN-6 | -0.5 | -10.2 | 3 | 0.800 | 0.086 | 4.89 | 0.05 | 39.1 | 48.53 | 11.59 |
| ORN-7 | 9.4 | 0 | 2.5 | 0.793 | 0.072 | 4.79 | 0.05 | 39.3 | 48.26 | 11.65 |
| ORN-8 | 20.1 | 9.8 | 2.5 | 0.894 | 0.076 | 7.15 | 0.07 | 38.7 | 49.20 | 11.47 |
| ORN-9 | 29.9 | 20.6 | 3 | 0.748 | 0.079 | 3.78 | 0.04 | 38.8 | 48.92 | 11.50 |
| ORN-10 | 39.7 | 30.5 | 2.5 | 0.921 | 0.088 | 3.61 | 0.04 | 38.6 | 49.10 | 11.44 |
| ORN-11 | 50 | 40.1 | 3 | 0.971 | 0.088 | 4.80 | 0.05 | 38.4 | 49.33 | 11.38 |
| ORN-12 | 60.2 | 50.5 | 3 | 1.023 | 0.087 | 3.54 | 0.04 | 39.3 | 48.26 | 11.65 |
| ORN-14 | 81 | 71.9 | 3 | 0.962 | 0.100 | 3.45 | 0.03 | 39.2 | 48.42 | 11.62 |
| ORN-16 | 100.9 | 91.4 | 3 | 1.033 | 0.120 | 3.59 | 0.04 | 39 | 48.49 | 11.56 |
| ORN-17 | 110.5 | 101.3 | 3 | 1.064 | 0.077 | 3.84 | 0.04 | 39.1 | 48.48 | 11.59 |
| ORN-18 | 120.8 | 111 | 2.5 | 0.962 | 0.072 | 3.89 | 0.04 | 39.2 | 48.36 | 11.62 |
| ORN-19 | 130.7 | 121.2 | 2.5 | 1.153 | 0.083 | 4.40 | 0.04 | 39.6 | 47.76 | 11.74 |
| ORN-20 | 140.6 | 131.1 | 3 | 1.261 | 0.127 | 4.16 | 0.04 | 39.6 | 47.85 | 11.74 |
| ORN-22 | 160.8 | 151.4 | 3 | 1.407 | 0.157 | 3.70 | 0.04 | 39.3 | 48.18 | 11.65 |
| ORN-23 | 171.3 | 161.3 | 3 | 1.204 | 0.145 | 4.36 | 0.04 | 39.1 | 48.45 | 11.59 |
| ORN-24 | 181.5 | 171.7 | 3 | 1.155 | 0.103 | 3.91 | 0.04 | 39.2 | 48.44 | 11.62 |
| ORN-25 | 191.7 | 182 | 3 | 1.382 | 0.160 | 4.46 | 0.04 | 39 | 48.58 | 11.56 |
| ORN-26 | 201.4 | 192.1 | 2.5 | 1.429 | 0.149 | 4.22 | 0.04 | 38.9 | 48.84 | 11.53 |
| ORN-27 | 211.4 | 201.9 | 3 | 1.245 | 0.103 | 4.49 | 0.04 | 39 | 48.57 | 11.56 |
| ORN-28 | 222 | 211.9 | 2 | 1.258 | 0.092 | 3.55 | 0.04 | 39.1 | 48.45 | 11.59 |
| ORN-29 | 231.9 | 222.6 | 3 | 1.343 | 0.112 | 3.54 | 0.04 | 38.9 | 48.64 | 11.53 |
| ORN-31 | 252.2 | 242.7 | 3 | 1.326 | 0.111 | 3.82 | 0.04 | 39.2 | 48.28 | 11.62 |
| ORN-32 | 262.3 | 252.6 | 3 | 1.366 | 0.099 | 3.50 | 0.03 | 39.2 | 48.18 | 11.62 |
| ORN-33 | 272.3 | 262.8 | 3 | 1.192 | 0.130 | 3.91 | 0.04 | 38.9 | 48.40 | 11.53 |
| ORN-34 | 282 | 272.9 | 3 | 1.364 | 0.111 | 3.38 | 0.03 | 38.7 | 48.58 | 11.47 |
| ORN-35 | 292 | 282.5 | 3 | 1.303 | 0.090 | 3.33 | 0.03 | 38.9 | 48.17 | 11.53 |
| ORN-36 | 301.9 | 292.5 | 3 | 1.431 | 0.128 | 3.11 | 0.03 | 38.8 | 48.06 | 11.50 |
| ORN-37 | 311.9 | 302.4 | 2 | 1.409 | 0.118 | 3.64 | 0.04 | 39.4 | 47.99 | 11.68 |
| ORN-38 | 322 | 312.4 | 3 | 1.445 | 0.097 | 3.39 | 0.03 | 39.3 | 48.20 | 11.65 |
| ORN-39 | 334 | 322.5 | 3 | 1.475 | 0.113 | 3.71 | 0.04 | 39.1 | 48.32 | 11.59 |
| ORN-40 | 342 | 332.5 | 3 | 1.161 | 0.112 | 3.60 | 0.04 | 38.9 | 48.64 | 11.53 |
| ORN-41 | 352.4 | 342.5 | 2.5 | 1.243 | 0.074 | 4.12 | 0.04 | 39.1 | 48.44 | 11.59 |
| ORN-42 | 361.9 | 352.9 | 3 | 1.538 | 0.163 | 3.52 | 0.04 | 39 | 48.60 | 11.56 |
| ORN-43a | 372 | 362.3 | 3 | 1.658 | 0.088 | 3.44 | 0.03 | 39.1 | 48.39 | 11.59 |
| ORN-44a | 382.3 | 372.5 | 3 | 1.437 | 0.082 | 3.59 | 0.04 | 38.5 | 49.20 | 11.41 |
| ORN-44b | 382.3 | 372.5 | 3 | 1.341 | 0.119 | 3.89 | 0.04 | 38.1 | 49.70 | 11.30 |
| ORN-45a | 392.5 | 383 | 3 | 1.315 | 0.132 | 3.37 | 0.03 | 38.2 | 49.69 | 11.33 |
| ORN-45b | 392.5 | 383 | 3 | 1.733 | 0.140 | 3.95 | 0.04 | 38.1 | 49.64 | 11.30 |
| ORN-46a | 403.1 | 393 | 3 | 1.467 | 0.078 | 3.29 | 0.03 | 38.5 | 49.26 | 11.41 |
| ORN-46b | 403.1 | 393 | 3 | 1.547 | 0.111 | 2.91 | 0.03 | 38.3 | 49.44 | 11.35 |
| ORN-47 | 412.1 | 403.6 | 3 | 1.428 | 0.077 | 3.03 | 0.03 | 38.1 | 49.70 | 11.30 |
| ORN-48 | 422.9 | 412.5 | 2.5 | 1.564 | 0.137 | 3.09 | 0.03 | 38.5 | 49.15 | 11.41 |
| ORN-49 | 433.8 | 423.3 | 3 | 1.761 | 0.159 | 3.88 | 0.04 | 38.5 | 49.11 | 11.41 |
| ORN-50 | 443.2 | 434.3 | 3 | 1.690 | 0.121 | 3.55 | 0.04 | 38.5 | 49.13 | 11.41 |
| ORN-51 | 453.7 | 443.8 | 3 | 1.390 | 0.101 | 3.49 | 0.03 | 38.4 | 49.35 | 11.38 |
| ORN-52 | 464.2 | 454.3 | 3 | 1.619 | 0.093 | 3.78 | 0.04 | 37.9 | 49.80 | 11.24 |
| ORN-53 | 474 | 464.6 | 2.5 | 1.341 | 0.116 | 4.40 | 0.04 | 38.1 | 49.62 | 11.30 |
| ORN-54 | 483.8 | 474.5 | 2.5 | 1.543 | 0.084 | 4.32 | 0.04 | 38.7 | 48.85 | 11.47 |
| ORN-55 | 493.2 | 484.3 | 3 | 1.472 | 0.097 | 4.00 | 0.04 | 38.6 | 49.20 | 11.44 |
| ORN-57 | 513.8 | 503.5 | 3 | 1.387 | 0.101 | 2.87 | 0.03 | 38.4 | 49.47 | 11.38 |
| ORN-59 | 534.8 | 523.8 | 3 | 1.416 | 0.082 | 2.97 | 0.03 | 38.4 | 49.24 | 11.38 |
| ORN-60 | 544.1 | 535.8 | 3 | 1.604 | 0.126 | 3.61 | 0.04 | 38.4 | 49.35 | 11.38 |
| ORN-61 | 553.3 | 544.6 | 3 | 1.527 | 0.087 | 3.24 | 0.03 | 38.5 | 49.26 | 11.41 |
| ORN-62 | 563.5 | 553.7 | 3 | 1.593 | 0.121 | 3.24 | 0.03 | 38.7 | 49.04 | 11.47 |
| ORN-63a | 573.6 | 564 | 2.5 | 1.493 | 0.094 | 3.62 | 0.04 | 38.2 | 49.71 | 11.33 |
| ORN-63 | 573.6 | 564 | 3 | 1.901 | 0.119 | 3.36 | 0.03 | 38 | 49.82 | 11.27 |
| ORN-64 | 583.3 | 574.2 | 3 | 1.298 | 0.134 | 4.09 | 0.04 | 38.7 | 49.23 | 11.47 |
| ORN-65 | 593.3 | 583.5 | 3 | 1.793 | 0.101 | 2.57 | 0.03 | 38.7 | 49.14 | 11.47 |
| ORN-66 | 603 | 593.9 | 3 | 1.690 | 0.082 | 2.32 | 0.02 | 39.9 | 47.62 | 11.83 |
| ORN-67 | 612.8 | 603.8 | 3 | 1.698 | 0.089 | 2.97 | 0.03 | 38.6 | 49.28 | 11.44 |
| ORN-68 | 622.8 | 613.5 | 3 | 1.722 | 0.087 | 3.94 | 0.04 | 39 | 48.79 | 11.56 |
| ORN-69 | 633.4 | 623.3 | 3 | 1.719 | 0.127 | 3.57 | 0.04 | 39.1 | 48.69 | 11.59 |
| ORN-70 | 643.8 | 633.8 | 2 | 1.835 | 0.093 | 3.15 | 0.03 | 39.1 | 48.69 | 11.59 |
| ORN-71 | 653.8 | 644.3 | 3 | 1.867 | 0.156 | 3.29 | 0.03 | 39.1 | 48.67 | 11.59 |
| ORN-72 | 663.8 | 654.2 | 3 | 1.673 | 0.121 | 3.21 | 0.03 | 39.1 | 48.53 | 11.59 |
| ORN-73 | 672.8 | 664.2 | 3 | 1.637 | 0.086 | 3.06 | 0.03 | 38.8 | 48.86 | 11.50 |
| ORN-74 | 683.7 | 674.5 | 3 | 1.766 | 0.151 | 3.98 | 0.04 | 38.9 | 48.71 | 11.53 |
| ORN-74b | 684 | 674.3 | 3 | 1.614 | 0.192 | 2.56 | 0.03 | 38.7 | 49.05 | 11.47 |
| ORN-75 | 697.2 | 684.8 | 3 | 1.919 | 0.127 | 3.15 | 0.03 | 38.9 | 48.67 | 11.53 |
| ORN-76 | 707.4 | 697.6 | 3 | 1.602 | 0.234 | 2.61 | 0.03 | 38.1 | 49.46 | 11.30 |
| ORN-77 | 716.7 | 708 | 3 | 1.612 | 0.106 | 3.18 | 0.03 | 38.1 | 49.75 | 11.30 |
| ORN-78 | 725.9 | 717.2 | 3 | 1.717 | 0.158 | 4.31 | 0.04 | 38.6 | 49.25 | 11.44 |
| ORN-79 | 740.8 | 726.3 | 3 | 1.792 | 0.090 | 3.27 | 0.03 | 38.6 | 49.23 | 11.44 |
| ORN-80 | 751.1 | 741.3 | 3 | 1.749 | 0.093 | 1.98 | 0.02 | 38.8 | 48.86 | 11.50 |
| ORN-81 | 760.9 | 751.6 | 2 | 1.866 | 0.138 | 1.96 | 0.02 | 39.4 | 48.29 | 11.68 |
| ORN-82 | 770.3 | 761.5 | 3 | 1.944 | 0.172 | 1.95 | 0.02 | 38.8 | 48.98 | 11.50 |
| ORN-83 | 779.9 | 770.8 | 3 | 1.940 | 0.074 | 2.20 | 0.02 | 38.8 | 48.96 | 11.50 |
| ORN-84 | 790.3 | 780.4 | 3 | 1.933 | 0.090 | 1.99 | 0.02 | 38.9 | 48.92 | 11.53 |
| ORN-85 | 799.5 | 790.5 | 3 | 2.137 | 0.100 | 1.81 | 0.02 | 38.6 | 49.26 | 11.44 |
| ORN-86 | 809.6 | 799.8 | 3 | 1.975 | 0.092 | 2.19 | 0.02 | 38.5 | 49.41 | 11.41 |
| ORN-87 | 819.7 | 810.4 | 3 | 2.080 | 0.100 | 2.24 | 0.02 | 38.8 | 48.96 | 11.50 |
| ORN-88 | 829.2 | 820.4 | 3 | 2.189 | 0.115 | 2.68 | 0.03 | 39 | 48.46 | 11.56 |
| ORN-89 | 839.5 | 829.9 | 3 | 2.140 | 0.134 | 3.73 | 0.04 | 39 | 48.60 | 11.56 |
| ORN-90 | 850.8 | 842.3 | 2.5 | 2.075 | 0.136 | 4.11 | 0.04 | 39.1 | 48.75 | 11.59 |
| ORN-91 | 861.6 | 851.6 | 3 | 1.983 | 0.150 | 3.80 | 0.04 | 39.2 | 48.50 | 11.62 |
| ORN-92 | 871.1 | 862.2 | 3 | 2.247 | 0.108 | 3.04 | 0.03 | 39.5 | 48.04 | 11.71 |
| ORN-93 | 881.1 | 870.8 | 3 | 2.145 | 0.108 | 2.41 | 0.02 | 38.8 | 48.94 | 11.50 |
| ORN-95 | 900.7 | 890.8 | 3 | 2.067 | 0.107 | 2.12 | 0.02 | 39 | 48.54 | 11.56 |
| ORN-96 | 911.1 | 901.2 | 3 | 2.280 | 0.096 | 2.21 | 0.02 | 39.3 | 48.16 | 11.65 |
| ORN-97 | 921.3 | 911.4 | 3 | 2.359 | 0.104 | 2.41 | 0.02 | 39 | 48.53 | 11.56 |
| ORN-99 | 942.2 | 931.9 | 3 | 2.313 | 0.207 | 2.99 | 0.03 | 38.6 | 49.13 | 11.44 |
| ORN-100 | 952.5 | 942.4 | 3 | 2.226 | 0.232 | 2.27 | 0.02 | 38.7 | 48.86 | 11.47 |
| ORN-101 | 961.8 | 952.9 | 2.5 | 2.319 | 0.216 | 2.44 | 0.02 | 38.5 | 49.05 | 11.41 |
| ORN-102 | 972.8 | 962.3 | 3 | 2.256 | 0.210 | 3.75 | 0.04 | 38.8 | 48.94 | 11.50 |
| ORN-103 | 982.1 | 973.3 | 3 | 2.762 | 0.099 | 3.11 | 0.03 | 38.8 | 48.90 | 11.50 |
| ORN-104 | 992.3 | 983 | 3 | 2.763 | 0.094 | 3.25 | 0.03 | 38.8 | 48.84 | 11.50 |
| ORN-105 | 1002.7 | 992.9 | 3 | 2.196 | 0.105 | 3.50 | 0.04 | 38.8 | 48.72 | 11.50 |
| ORN-106 | 1012.3 | 1003.2 | 3 | 2.625 | 0.144 | 4.32 | 0.04 | 39.3 | 48.26 | 11.65 |
| ORN-107a | 1022.6 | 1014.3 | 3 | 2.669 | 0.121 | 3.88 | 0.04 | 39.4 | 48.21 | 11.68 |
| ORN-107 | 1022.8 | 1013 | 3 | 2.422 | 0.087 | 4.40 | 0.04 | 39.6 | 48.13 | 11.74 |
| ORN-108 | 1031.9 | 1022.8 | 3 | 2.951 | 0.127 | 3.72 | 0.04 | 39 | 48.62 | 11.56 |
| ORN-109 | 1041.4 | 1032.7 | 3 | 2.904 | 0.126 | 5.09 | 0.05 | 39.5 | 48.14 | 11.71 |
| ORN-111 | 1061.9 | 1053.2 | 3 | 2.883 | 0.091 | 3.71 | 0.04 | 39.4 | 48.05 | 11.68 |
| ORN-112 | 1071.7 | 1062.5 | 3 | 2.945 | 0.118 | 4.42 | 0.04 | 39.3 | 48.14 | 11.65 |
| ORN-113 | 1082.6 | 1072.6 | 3 | 3.155 | 0.105 | 5.78 | 0.06 | 39.8 | 47.82 | 11.80 |
| ORN-114 | 1101.1 | 1091.4 | 3 | 3.305 | 0.114 | 4.52 | 0.05 | 39.7 | 47.83 | 11.77 |
| ORN-115 | 1110.7 | 1101.7 | 3 | 3.238 | 0.112 | 5.02 | 0.05 | 39.9 | 47.68 | 11.83 |
| ORN-116 | 1128.6 | 1110.9 | 3 | 3.264 | 0.118 | 5.55 | 0.06 | 39.7 | 47.92 | 11.77 |
| ORN-117 | 1127.9 | 1118.9 | 3 | 4.039 | 0.262 | 5.33 | 0.05 | 39.4 | 48.34 | 11.68 |
| ORN-118 | 1139.5 | 1128.8 | 3 | 3.821 | 0.226 | 6.11 | 0.06 | 39.4 | 48.18 | 11.68 |
| ORN-119 | 1150 | 1140 | 2.5 | 3.564 | 0.109 | 3.74 | 0.04 | 39 | 48.38 | 11.56 |
| ORN-120 | 1160.7 | 1150.6 | 3 | 3.562 | 0.137 | 4.79 | 0.05 | 38.9 | 48.78 | 11.53 |
| * Measured with accelerator mass spectrometry (AMS).  ✝Measured with ICP in Actlabs Analytical services, Canada. | | | | | | | |  |  |  |

Table A4. Blank measurements along with associated samples processed in similar batches

| Blank name | Sample names | ^36^Cl/^35^Cl final  (10^-12^) | | Error (10^-12^) | ^37^Cl/^35^Cl  (%) | Error (%) |
| --- | --- | --- | --- | --- | --- | --- |
| BL-CL-59 | ORN1 to 13 | | -0.0006 | 0.0005 | 0.27 | 0.018 |
| Bl-Cl-60 | ORN14 to 28 | | -0.0011 | 0.0000 | 0.27 | 0.018 |
| Bl-Cl-62 | ORN29 to 43a | | -0.0005 | 0.0005 | 0.29 | 0.009 |
| Bl-Cl-63 | ORN43b to 54 | | -0.0001 | 0.0009 | 0.31 | 0.011 |
| BL-CL-64 | ORN55 to 68 | | -0.0001 | 0.0016 | 0.27 | 0.007 |
| Bl-Cl-65 | ORN69 to 82 | | -0.0001 | 0.0016 | 0.29 | 0.015 |
| BL-CL-66 | ORN83 to 97 | | -0.0010 | 0.0004 | 0.29 | 0.003 |
| BL-CL-69 | ORN98 to111 | | -0.0011 | 0.0000 | 0.30 | 0.010 |
| BL-CL-70 | ORN112 to 120; RHM1 to 6 | | 0.0024 | 0.0018 | 0.32 | 0.003 |
| BL-CL-71 | RHM7 to 21 | | 0.0009 | 0.0012 | 0.31 | 0.003 |
| BL-CL-72 | RHM22 to 35 | | 0.0013 | 0.0012 | 0.32 | 0.007 |
| BL-CL-73 | RHM36 to 50 | | 0.0019 | 0.0018 | 0.31 | 0.005 |
| BL-CL-74 | RHM51a to 63a | | 0.0002 | 0.0012 | 0.33 | 0.001 |
| BL-CL-75 | RHM63b to 74b | | -0.0003 | 0.0011 | 0.33 | 0.004 |
| BL-CL-78 | RHM75 to 80 | | 0.0003 | 0.0027 | 0.30 | 0.002 |
| *Note:* Blanks corrected based on average of all the blanks | | | | | | |

Table A5. Mean chemical composition of the Rahmiye fault scarp samples and colluvium

| Sample name | Cl  (%) | O  (%) | C  (%) | CaO  (%) | MgO  (%) | Al_2_O_3_  (%) | SiO_2_  (%) | P_2_O_5_  (%) | K_2_O  (%) |
| --- | --- | --- | --- | --- | --- | --- | --- | --- | --- |
| RHM-2 | 0.0001 | 49.18 | 11.53 | 55.37 | 0.03 | 0.10 | 0.005 | 0.03 | 0.010 |
| RHM-11 | 0.0002 | 48.99 | 11.56 | 55.28 | 0.03 | 0.13 | 0.005 | 0.02 | 0.010 |
| RHM-16 | 0.0003 | 48.95 | 11.56 | 55.58 | 0.03 | 0.12 | 0.005 | 0.01 | 0.010 |
| RHM-28 | 0.0001 | 49.13 | 11.53 | 55.07 | 0.04 | 0.15 | 0.005 | 0.02 | 0.020 |
| RHM-39 | 0.0002 | 48.33 | 11.71 | 55.31 | 0.02 | 0.11 | 0.005 | 0.03 | 0.005 |
| RHM-51b | 0.0002 | 48.37 | 11.68 | 55.12 | 0.04 | 0.16 | 0.005 | 0.03 | 0.020 |
| RHM-62 | 0.0022 | 50.13 | 11.06 | 53.34 | 0.03 | 0.13 | 0.005 | 0.01 | 0.010 |
| RHM-71b | 0.0077 | 51.07 | 10.52 | 51.07 | 0.04 | 0.18 | 0.005 | 0.03 | 0.040 |
| RHM-80 | 0.0002 | 48.85 | 11.59 | 55.48 | 0.03 | 0.09 | 0.005 | 0.01 | 0.010 |
| Average | 0.0015 | 49.68 | 11.29 | 54.62 | 0.03 | 0.13 | 0.005 | 0.02 | 0.015 |
|  | TiO_2_  (%) | MnO  (%) | Fe_2_O_3_  (%) | B  (ppm) | Sm  (ppm) | Gd  (ppm) | U  (ppm) | Th  (ppm) |  |
| RHM-2 | 0.001 | 0.004 | 0.05 | 2 | 0.2 | 0.2 | 0.3 | 0.1 |  |
| RHM-11 | 0.002 | 0.003 | 0.06 | 3 | 0.2 | 0.2 | 0.3 | 0.1 |  |
| RHM-16 | 0.002 | 0.004 | 0.05 | 1 | 0.2 | 0.2 | 0.3 | 0.1 |  |
| RHM-28 | 0.002 | 0.007 | 0.09 | 1 | 0.2 | 0.3 | 0.3 | 0.1 |  |
| RHM-39 | 0.003 | 0.006 | 0.06 | 1 | 0.2 | 0.2 | 0.4 | 0.1 |  |
| RHM-51b | 0.003 | 0.004 | 0.06 | 4 | 0.3 | 0.3 | 0.3 | 0.1 |  |
| RHM-62 | 0.003 | 0.005 | 0.06 | 1 | 0.3 | 0.3 | 0.3 | 0.1 |  |
| RHM-71b | 0.005 | 0.005 | 0.08 | 1 | 0.4 | 0.4 | 0.5 | 0.1 |  |
| RHM-80 | 0.002 | 0.004 | 0.05 | 1 | 0.2 | 0.3 | 0.4 | 0.1 |  |
| Average | 0.002 | 0.005 | 0.06 | 2 | 0.2 | 0.3 | 0.3 | 0.1 |  |

Table A6. Mean chemical composition of the Ören fault scarp samples and colluvium

| Sample name | Cl  (%) | O  (%) | C  (%) | CaO  (%) | MgO  (%) | Al_2_O_3_  (%) | SiO_2_  (%) | P_2_O_5_  (%) | K_2_O  (%) |
| --- | --- | --- | --- | --- | --- | --- | --- | --- | --- |
| ORN-2 | 0.0004 | 48.76 | 11.53 | 54.87 | 0.03 | 0.28 | 0.450 | 0.03 | 0.080 |
| ORN-12 | 0.0004 | 48.25 | 11.65 | 55.07 | 0.03 | 0.28 | 0.390 | 0.04 | 0.070 |
| ORN-23 | 0.0004 | 48.45 | 11.59 | 54.41 | 0.03 | 0.32 | 0.400 | 0.01 | 0.100 |
| ORN-34 | 0.0003 | 48.57 | 11.47 | 54.88 | 0.03 | 0.55 | 0.750 | 0.01 | 0.160 |
| ORN-45a | 0.0003 | 49.69 | 11.32 | 55.23 | 0.03 | 0.34 | 0.400 | 0.04 | 0.100 |
| ORN-57 | 0.0003 | 49.47 | 11.38 | 54.45 | 0.03 | 0.29 | 0.250 | 0.02 | 0.080 |
| ORN-67 | 0.0003 | 49.28 | 11.44 | 54.66 | 0.03 | 0.28 | 0.340 | 0.02 | 0.080 |
| ORN-76 | 0.0003 | 49.46 | 11.29 | 54.36 | 0.04 | 0.44 | 0.630 | 0.05 | 0.130 |
| ORN-86 | 0.0002 | 49.41 | 11.41 | 54.99 | 0.03 | 0.29 | 0.300 | 0.03 | 0.090 |
| ORN-95 | 0.0002 | 48.53 | 11.56 | 55.13 | 0.03 | 0.35 | 0.430 | 0.04 | 0.110 |
| ORN-104 | 0.0003 | 48.84 | 11.50 | 54.62 | 0.04 | 0.34 | 0.550 | 0.05 | 0.090 |
| ORN-112 | 0.0004 | 48.14 | 11.65 | 54.85 | 0.03 | 0.25 | 0.720 | 0.02 | 0.060 |
| ORN-120 | 0.0005 | 48.78 | 11.53 | 55.22 | 0.03 | 0.23 | 0.290 | 0.005 | 0.060 |
| Average | 0.0004 | 48.74 | 11.53 | 54.83 | 0.03 | 0.33 | 0.454 | 0.03 | 0.093 |
|  | TiO_2_  (%) | MnO  (%) | Fe_2_O_3_  (%) | B  (ppm) | Sm  (ppm) | Gd  (ppm) | U  (ppm) | Th  (ppm) |  |
| ORN-2 | 0.011 | 0.008 | 0.15 | 4 | 0.4 | 0.5 | 1.3 | 0.2 |  |
| ORN-12 | 0.01 | 0.007 | 0.17 | 1 | 0.4 | 0.5 | 1.5 | 0.2 |  |
| ORN-23 | 0.013 | 0.006 | 0.15 | 5 | 0.3 | 0.5 | 3.3 | 0.2 |  |
| ORN-34 | 0.026 | 0.006 | 0.25 | 3 | 0.5 | 0.7 | 2.4 | 0.3 |  |
| ORN-45a | 0.014 | 0.005 | 0.14 | 5 | 0.6 | 0.7 | 2.0 | 0.2 |  |
| ORN-57 | 0.011 | 0.006 | 0.12 | 6 | 0.4 | 0.5 | 1.6 | 0.2 |  |
| ORN-67 | 0.011 | 0.007 | 0.12 | 7 | 0.5 | 0.6 | 1.7 | 0.2 |  |
| ORN-76 | 0.018 | 0.006 | 0.16 | 5 | 0.7 | 0.8 | 1.7 | 0.2 |  |
| ORN-86 | 0.011 | 0.008 | 0.12 | 6 | 0.5 | 0.6 | 1.6 | 0.05 |  |
| ORN-95 | 0.014 | 0.006 | 0.16 | 5 | 0.3 | 0.3 | 1.4 | 0.2 |  |
| ORN-104 | 0.013 | 0.006 | 0.14 | 2 | 0.5 | 0.5 | 1.1 | 0.3 |  |
| ORN-112 | 0.009 | 0.008 | 0.11 | 2 | 0.3 | 0.4 | 0.7 | 0.3 |  |
| ORN-120 | 0.008 | 0.008 | 0.12 | 3 | 0.3 | 0.3 | 0.7 | 0.2 |  |
| Average | 0.013 | 0.007 | 0.15 | 4 | 0.4 | 0.5 | 1.6 | 0.2 |  |

Table A7. Output results of the lowest statistical criterion for different rupture histories of the faults

| Fault | Event number | Beginning of ^36^Cl accumulation (ka) | Age (ka) | Slip (m) | statistical criteria |
| --- | --- | --- | --- | --- | --- |
| Rahmiye | 4 | 57 | 11.4  11.0  3.0  2.7 | 1.9  1.0  1.7  1.8 | Χ^2^ = 1.9  RMSw = 1.3  AICc = 235 |
|  | 5 | 48 | 12.0  11.3  10.0  4.8  3.9 | 0.8  1.9  0.2  0.4  2.9 | Χ^2^ = 2.19  RMSw = 1.3  AICc = 268 |
| Ören | 3 | 25 | 8.6  5.5  2.0 | 3.7  6.1  2.1 | Χ^2^ = 1.5  RMSw = 1.2  AICc = 470 |
|  | 5 | 24 | 9.9  9.0  6.2  4.4  1.7 | 0.4  2.9  4.1  2.4  1.7 | Χ^2^ = 1.6  RMSw = 1.1  AICc = 488 |
|  | 6 | 29 | 7.7  5.4  4.2  3.5  2.2  1.8 | 4.0  5.2  0.4  0.03  0.4  2.0 | Χ^2^ = 1.5  RMSw = 1.2  AICc = 480 |
